# Multi-habitat microbiome profiling identifies habitat-dependent alterations and complementary discriminatory information in urolithiasis

**DOI:** 10.3389/fcimb.2026.1853826

**Published:** 2026-07-16

**Authors:** Zhentao Jia, Yujiao Peng, Shuangjian Jiang, Yiming Tang, Hening Yin, Lijuan Huang, Pengju Li, Chengqiang Mo, Rongpei Wu

**Affiliations:** 1 Department of Urology, The First Affiliated Hospital, Sun Yat-Sen University, Guangzhou, China; 2 Department of Orthopedic Surgery, The First Affiliated Hospital, Sun Yat-Sen University, Guangzhou, China; 3 Department of Urology, The Second Affiliated Hospital, Guangzhou Medical University, Guangzhou, China

**Keywords:** 16S rRNA gene sequencing, fecal microbiome, habitat-specific alterations, multi-habitat profiling, random forest, salivary microbiome, urinary microbiome, urolithiasis

## Abstract

**Background:**

Urolithiasis has been associated with microbial alterations in individual anatomical niches; however, whether microbial signatures across urinary, intestinal, and oral habitats represent shared, site-specific, or complementary disease-associated information remains unclear. This study aimed to characterize multi-habitat microbiome alterations associated with urolithiasis and to evaluate whether integrated multi-site profiling captures internally cross-validated disease-associated microbial information.

**Methods:**

Salivary, clean-catch midstream urinary, and fecal samples were collected from 80 stone formers (SF) and 40 healthy controls (HC) and profiled using 16S rRNA gene sequencing. After quality control, the final analytical dataset comprised 101 urinary, 117 fecal, and 120 salivary samples, with 98 participants contributing complete three-habitat profiles. Habitat-specific alpha- and beta-diversity, taxonomic alterations, and exploratory inferred-network and predicted-functional profiles were evaluated. Random forest models were assessed using repeated nested stratified cross-validation to examine internal discriminatory information from single- and multi-habitat microbial features.

**Results:**

Urolithiasis was associated with statistically detectable but modest differences in microbial community structure across all three habitats, with small PERMANOVA effect sizes and significant dispersion differences. Fecal samples from SF showed significantly reduced richness, including lower Sobs, Chao1, and ACE indices than HC after false discovery rate correction (all q = 0.022), whereas urinary and salivary alpha-diversity did not show broad loss. Taxonomic alterations were habitat dependent: saliva yielded the broadest covariate-robust genus-level candidate set, feces showed fewer stable HC-enriched genera alongside reduced richness, and urinary candidate associations were identified but require prospective contamination-controlled validation because of the low-biomass nature of urine and the absence of negative controls. In matched participants, the combined multi-habitat microbiome model achieved an area under the receiver operating characteristic curve of 0.865 (95% CI, 0.839-0.886), exceeding the limited clinical-only model based on age, sex, and body mass index (AUC, 0.738; delta-AUC, 0.128; 95% CI, 0.026-0.229). Improvement over the best single-habitat microbiome model was not statistically conclusive. External contextual analyses provided partial urinary community-level support in KiSMi and an inverse but FDR-non-significant NHANES oral-richness association after extensive covariate adjustment, supporting harmonized prospective validation.

**Conclusions:**

These findings identify habitat-dependent microbiome alterations in urolithiasis and show that integrated multi-habitat profiling captures disease-associated microbial information beyond a limited clinical baseline. The combination of reduced fecal richness, broad salivary covariate-robust candidates, biologically proximal urinary candidates, and external contextual signals supports simultaneous multi-site profiling as a valuable framework for future mechanistic and translational studies. Prospective studies with rigorous low-biomass controls, comprehensive exposure metadata, direct functional measurements, and prespecified independent validation are warranted.

## Introduction

Urolithiasis is one of the most common disorders of the urinary tract and is characterized by a high tendency to recur (Skolarikos et al., 2024). Stone formation reflects interactions among genetic susceptibility, urinary supersaturation, abnormal mineral handling, urine pH, diet, fluid intake, metabolic status, environmental exposure, and local urinary tract conditions (Bargagli et al., 2025; Cao et al., 2025). Although contemporary endoscopic and minimally invasive procedures have improved stone clearance, they do not directly resolve the biological processes that predispose patients to recurrent disease (Skolarikos et al., 2024; Shpitzer et al., 2025). The continuing burden of recurrence underscores the need to better define biological factors that may contribute to stone formation, persistence, and patient heterogeneity (Han et al., 2025).

Traditional models of urolithiasis emphasize physicochemical and host metabolic processes such as hyperoxaluria, hypercalciuria, hypocitraturia, hyperuricosuria, and urinary pH abnormalities (Shastri et al., 2023). These mechanisms remain fundamental, but they do not fully explain why patients with apparently similar metabolic profiles can differ in stone phenotype, recurrence risk, and response to preventive interventions (Hsi et al., 2024; Bargagli et al., 2025). Microbiome research offers an additional ecological perspective. Intestinal microbes may influence oxalate handling, short-chain fatty acid production, bile-acid metabolism, epithelial barrier function, and systemic inflammatory tone (Ticinesi et al., 2018; Jia et al., 2025; Hou et al., 2025; Li D. et al., 2025; Li X.-M. et al., 2025; Liu L. et al., 2025; Chen et al., 2026; Yang, 2025). The urinary tract is now recognized to contain measurable microbial communities rather than being uniformly sterile, and urinary microbial profiles may be relevant to local inflammation, biofilm formation, and the microenvironment surrounding stones (Agudelo et al., 2024; Chorbińska et al., 2025; Reasoner et al., 2025; Wu et al., 2025; Zhang J. et al., 2025; Zhang et al., 2026). Oral and salivary microbial communities may also capture mucosal, dietary, inflammatory, and systemic metabolic information that is not represented by gut or urinary sampling alone (Xu et al., 2024; Lu et al., 2025; Zhang Z. et al., 2025).

These observations motivate a multi-habitat sampling framework and leave unresolved how urinary, intestinal, and oral microbial signals relate to one another in stone disease. Most previous studies have focused on one habitat or have compared sites across different cohorts, making it difficult to determine whether stone-associated microbial alterations are local, shared, or technically context-dependent (Al et al., 2023; Kunath et al., 2024; Jia et al., 2025). A unified three-habitat design within the same participants can address three related questions: (i) whether urolithiasis is associated with microbiome differences within each habitat; (ii) whether these differences are habitat dependent rather than a single cross-site phenomenon; and (iii) whether multi-habitat profiles capture microbial information beyond basic clinical variables such as age, sex, and BMI.

We therefore profiled urinary, fecal, and salivary microbiota from patients with urolithiasis, hereafter referred to as stone formers (SF), and healthy controls (HC) within a common 16S rRNA gene sequencing workflow. We hypothesized that urolithiasis would be associated with habitat-dependent microbiome alterations across the three sampling sites. We further evaluated whether integrated multi-habitat profiling could provide discriminatory information beyond single-habitat or limited clinical features, while treating inferred-network, predicted-functional, clinical-association, stone-composition, cross-habitat, and external public-dataset analyses as exploratory or contextual extensions.

## Results

### Study design and final analytical cohort

The study enrolled 120 participants, including 80 patients with urolithiasis and 40 healthy controls. Saliva, clean-catch midstream urine, and fecal samples were collected under fasting conditions and processed using a common 16S rRNA gene sequencing workflow. After sample-level quality control and rarefaction eligibility filtering, the final internal analytic dataset included 101 urinary samples, 117 fecal samples, and 120 salivary samples. The urinary analysis set comprised 68 cases and 33 controls, the fecal analysis set comprised 79 cases and 38 controls, and the salivary analysis set comprised all 80 cases and 40 controls. Ninety-eight participants, including 67 cases and 31 controls, contributed complete urinary, fecal, and salivary profiles for matched multi-habitat analyses (**Table 1**; **Supplementary Figure 4**).

**Table 1:** Baseline characteristics and final sample availability in the internal cohort.

Characteristic	SF (n=80)	HC (n=40)	P value
Participants, n	80	40	–
Female, n (%)	25 (31.2)	2 (5.0)	<0.001
Age, years	53.5 (41.8-62.0)	42.5 (27.0-63.0)	0.028
BMI, kg/m2	24.2 (22.0-27.0)	22.7 (20.1-26.5)	0.144
Serum calcium, mmol/L	2.2 (2.1-2.2)	2.2 (2.1-2.3)	0.361
Serum uric acid, umol/L	319.0 (256.8-392.5)	327.0 (258.8-353.5)	0.670
Serum creatinine, umol/L	78.5 (67.2-98.0)	77.5 (72.8-86.2)	0.841
Maximum stone dimension, mm	13.0 (9.0-16.0)	Not applicable	–
Stone composition	Calcium oxalate (CaOx), n=58; Calcium phosphate (CaP), n=14; Uric acid (UA), n=8	Not applicable	–
Urinary samples retained, n	68	33	–
Fecal samples retained, n	79	38	–
Salivary samples retained, n	80	40	–
Participants with all three habitats, n	67	31	–

For alpha- and beta-diversity analyses, eligible internal samples were rarefied to a fixed depth of 22,254 reads per sample. Age, sex, and BMI were available in the internal metadata and were used as core covariates in sensitivity analyses. Because the cohort differed in age and sex distribution between SF and HC, disease-associated signals were interpreted alongside covariate-adjusted sensitivity analyses where applicable; these analyses cannot eliminate residual confounding in an observational cohort. Diet, habitual fluid intake, non-antibiotic medication exposure such as NSAID or antispasmodic use, and socioeconomic variables were not available as reliable analyzable internal fields. These variables were therefore handled as sources of residual confounding rather than incorporated into the core models. The reporting structure prioritized three levels of evidence: primary habitat-specific community and taxonomic findings; internal discriminatory modeling using matched multi-habitat samples; and exploratory analyses designed to generate hypotheses rather than establish mechanism. Sample retention, sequencing-depth distributions, rarefaction eligibility, Good’s coverage, and covariate missingness are summarized in **Supplementary Figure 4** and **Supplementary Table 1**.

### Urolithiasis was associated with habitat-dependent differences in microbial community distributions

**Figure 1:**
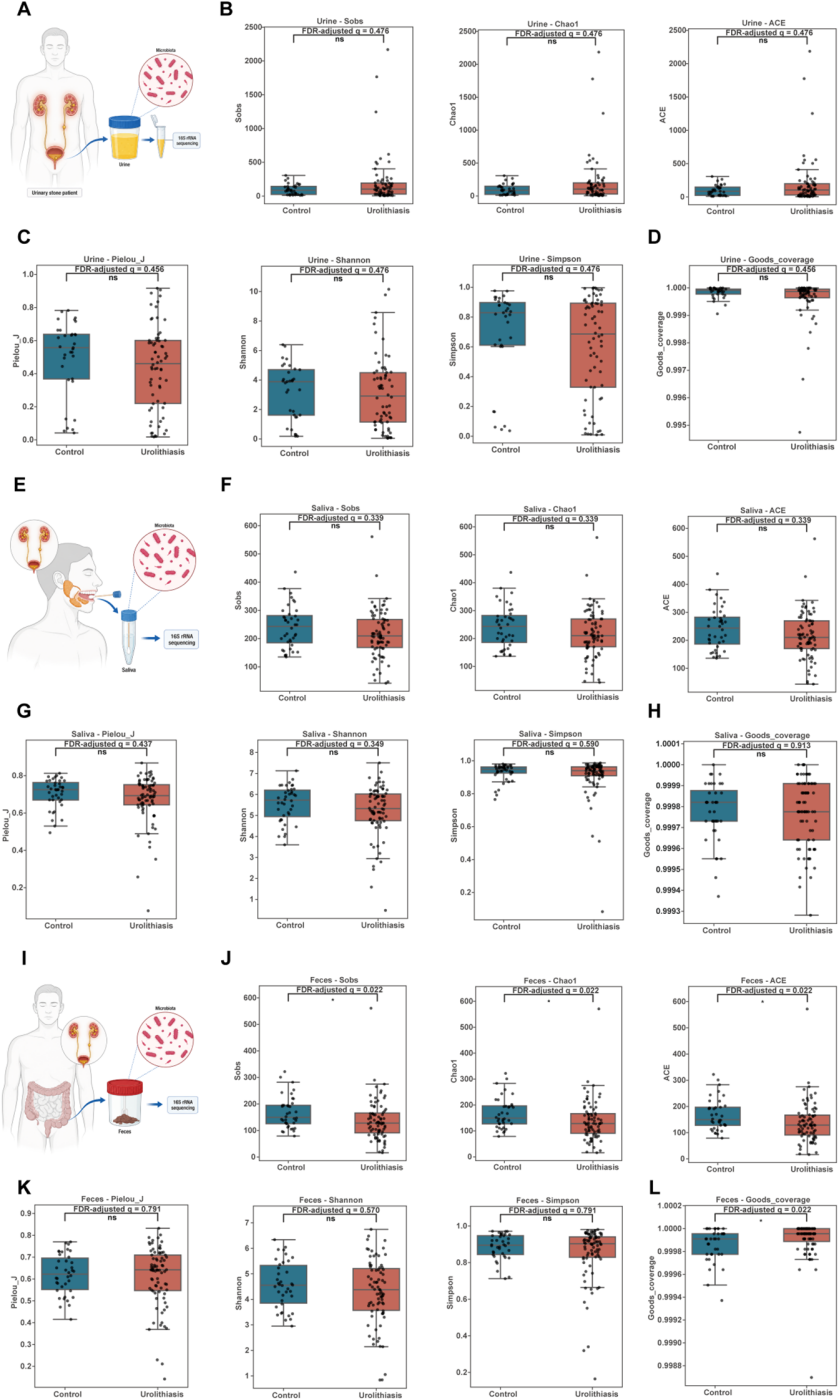
Alpha-diversity profiles across urinary, salivary, and fecal microbiota in stone formers and healthy controls. **(A)** Schematic overview of urinary sample collection and 16S rRNA gene sequencing. **(B–D)** Urinary alpha-diversity metrics, including **(B)** richness indices (Sobs, Chao1, and ACE), **(C)** diversity/evenness indices (Pielou’s evenness, Shannon, and Simpson), and **(D)** Good’s coverage. **(E)** Schematic overview of salivary sample collection. **(F–H)** Salivary alpha-diversity metrics, including **(F)** richness indices, **(G)** diversity/evenness indices, and **(H)** Good’s coverage. **(I)** Schematic overview of fecal sample collection. **(J–L)** Fecal alpha-diversity metrics, including **(J)** richness indices, **(K)** diversity/evenness indices, and **(L)** Good’s coverage. Group comparisons were performed separately within each habitat using two-sided Mann-Whitney U tests followed by Benjamini-Hochberg false-discovery rate (FDR) correction. Exact FDR-adjusted q values are shown in the corresponding panels. SF, stone formers; HC, healthy controls; ns, not significant; *q < 0.05.

Alpha-diversity results showed a habitat-dependent pattern rather than a uniform multi-site diversity loss (**Figure 1**). In urinary samples, no richness, diversity, evenness, or coverage metric differed significantly between cases and controls after within-habitat FDR correction. Salivary samples showed a similar pattern, with no FDR-significant case-control differences for Sobs, Chao1, ACE, Shannon, Simpson, Pielou’s evenness, or Good’s coverage.

Fecal samples showed the clearest alpha-diversity signal. Richness-related indices were lower in SF than in HC within the fecal habitat: median Sobs was 128.0 versus 149.5 (FDR-adjusted q = 0.022), median Chao1 was 128.7 versus 149.5 (FDR-adjusted q = 0.022), and median ACE was 129.5 versus 149.8 (FDR-adjusted q = 0.022). These richness metrics remained significant after within-habitat FDR correction and retained concordant directions in age/sex/BMI-adjusted sensitivity models. A male-restricted sensitivity analysis retained the fecal richness signal for Sobs, Chao1, and ACE after FDR correction, indicating that this finding persisted under sex-restricted analysis, although residual confounding cannot be excluded. Complete alpha-diversity results across all seven metrics, global FDR sensitivity, age/sex/BMI-adjusted rank-based models, and male-restricted sensitivity analyses are shown in **Supplementary Figure 5** and **Supplementary Table 2**.

**Figure 2:**
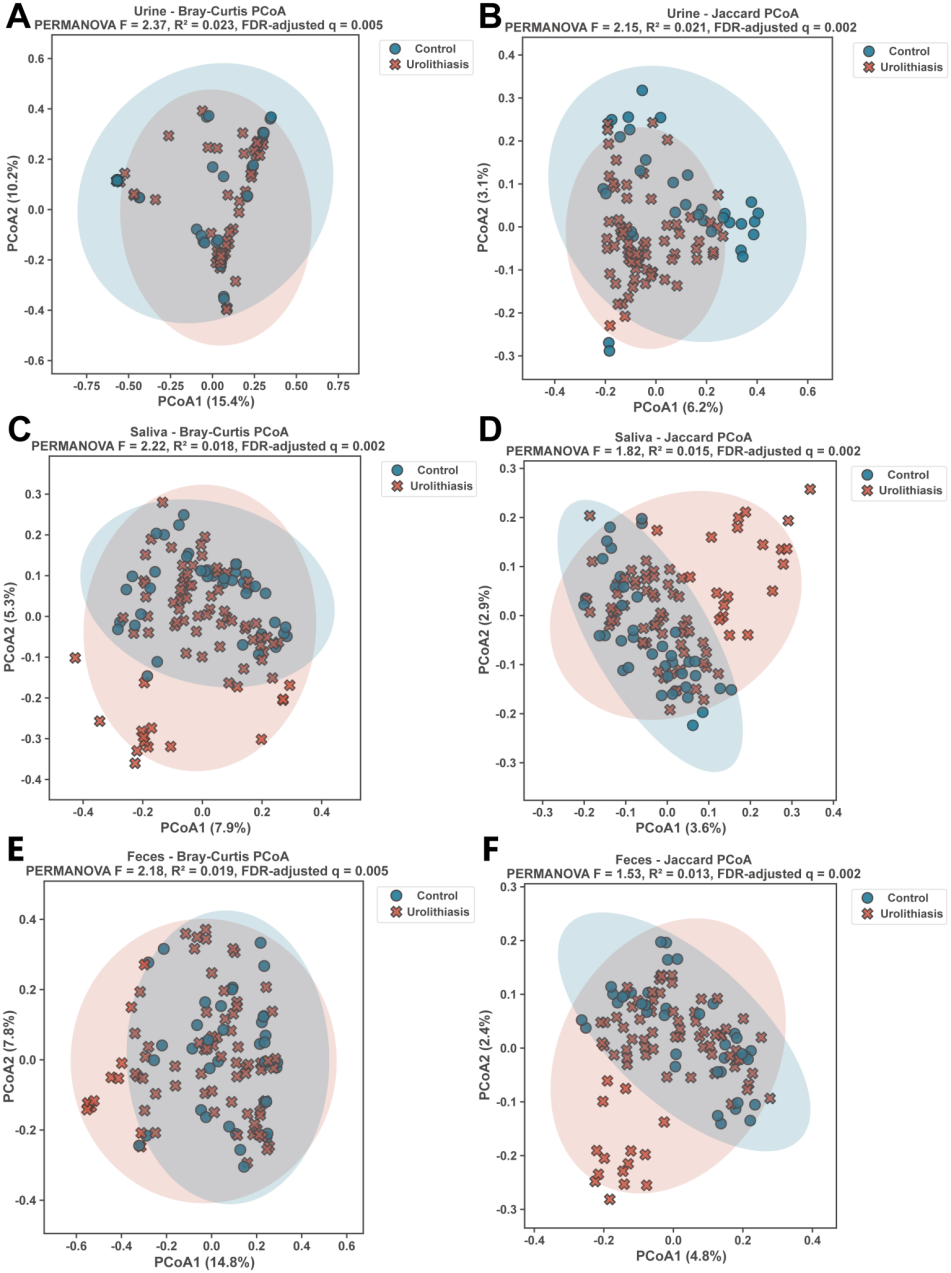
Beta-diversity ordination of urinary, salivary, and fecal microbial communities. **(A, B)** Principal coordinates analysis (PCoA) of urinary microbiota based on **(A)** Bray-Curtis and **(B)** Jaccard distances. **(C, D)** PCoA of salivary microbiota based on **(C)** Bray-Curtis and **(D)** Jaccard distances. **(E, F)** PCoA of fecal microbiota based on **(E)** Bray-Curtis and **(F)** Jaccard distances. Ellipses denote 95% confidence regions for each group. PERMANOVA F statistics, R2 values, and FDR-adjusted q values are displayed in the panels. Because PERMDISP was significant in the corresponding comparisons, these ordinations are interpreted as statistically detectable but modest community-distribution differences with a dispersion component, rather than complete separation between SF and HC. SF, stone formers; HC, healthy controls; PCoA, principal coordinates analysis.

Beta-diversity analyses identified statistically detectable but modest case-control differences in community distributions across all three habitats (**Figure 2**). Using Bray-Curtis distance, PERMANOVA q values were 0.005 for urine, 0.005 for feces, and 0.002 for saliva, with corresponding R2 values of 0.022, 0.016, and 0.023. Jaccard-based PERMANOVA produced similar evidence of group-associated community-distribution differences, with R2 values of 0.013-0.020. The stronger Bray-Curtis than Jaccard signals are consistent with case-control differences being more apparent in relative-abundance structure than in simple presence-absence patterns. Across habitats, the small effect sizes should not be interpreted as ranking the magnitude of alteration between specimen types.

PERMDISP was also significant for the corresponding internal beta-diversity comparisons, and distance-to-centroid sensitivity plots showed greater within-group dispersion among SF across habitats and distance metrics. Because PERMDISP was significant for the corresponding internal comparisons, these beta-diversity findings were interpreted as modest community-distribution differences that included a within-group dispersion component rather than complete separation between SF and HC. Distance-to-centroid analyses are shown in **Supplementary Figure 3**, and full PERMANOVA/PERMDISP outputs are provided in **Supplementary Table 3**.

### Stable taxonomic alterations differed across habitats

**Figure 3:**
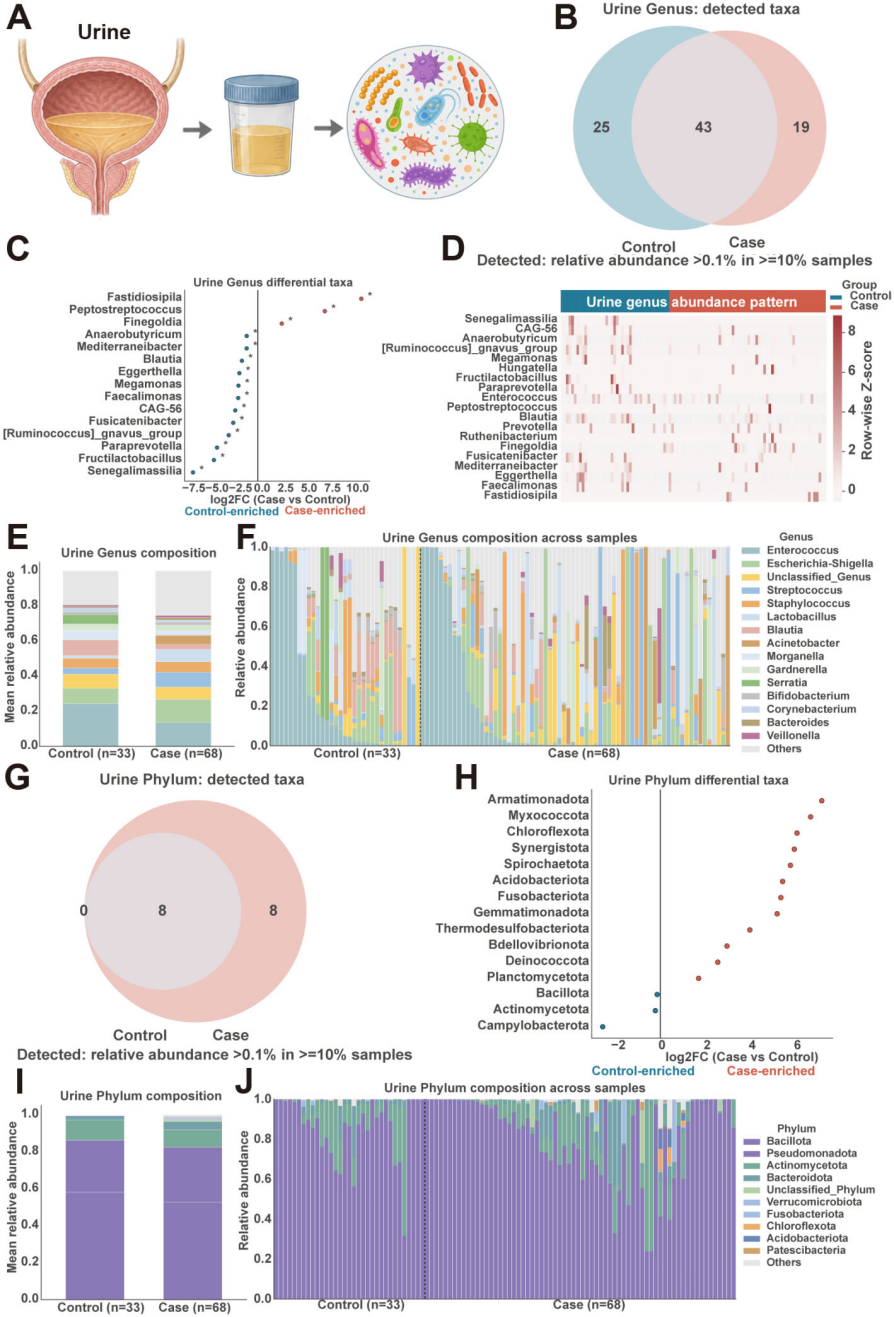
Urinary taxonomic composition and candidate microbial alterations associated with urolithiasis. **(A)** Workflow schematic for urinary microbiome profiling by 16S rRNA gene sequencing. **(B)** Venn diagram summarizing detected urinary genera in SF and HC, using the detection criterion of relative abundance >0.1% in at least 10% of samples within the corresponding group. **(C)** Differential urinary genera displayed as log2 fold change for SF versus HC; positive values indicate SF enrichment and negative values indicate HC enrichment. Primary FDR-adjusted q values are shown. **(D)** Genus-level row-wise Z-score heatmap across individual urinary samples. **(E, F)** Urinary genus-level composition summarized at group-mean and individual-sample levels. **(G–I)** Phylum-level urinary taxonomic summaries, including detected phyla, differential phyla, and relative-abundance composition. Differential testing used two-sided Mann-Whitney U tests with Benjamini-Hochberg FDR correction within the urinary taxonomic level. Urinary genus-level associations should be interpreted as preliminary candidates because urine is a low-biomass specimen type and matched extraction or PCR negative controls were not available for retrospective contamination assessment. SF, stone formers; HC, healthy controls; FDR, false-discovery rate.

**Figure 4:**
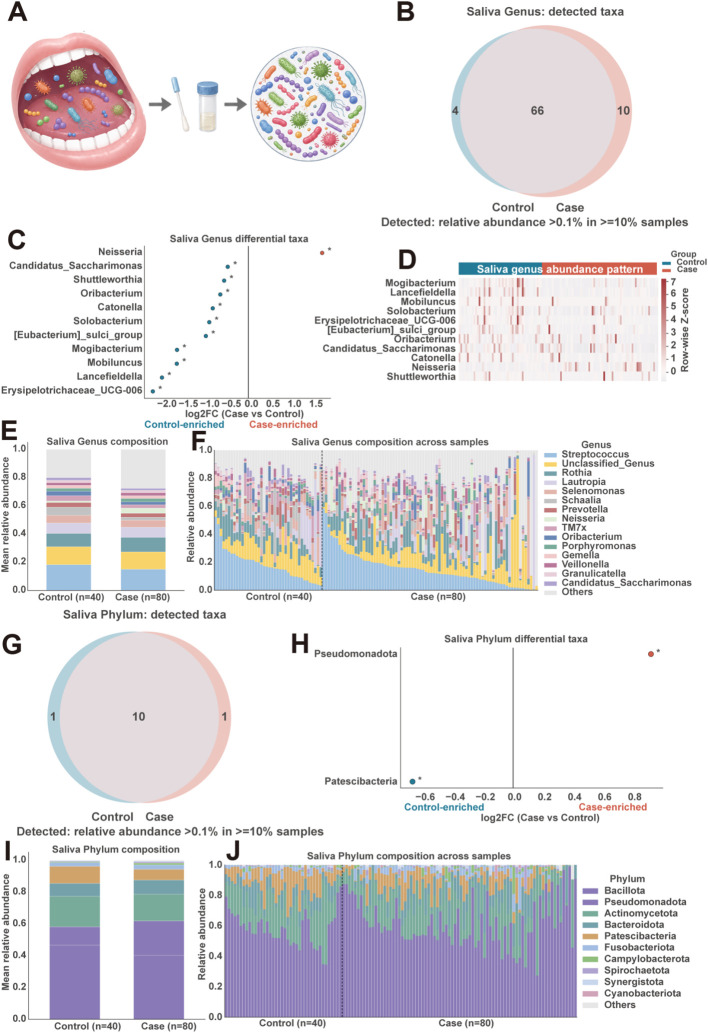
Salivary taxonomic composition and candidate microbial alterations associated with urolithiasis. **(A)** Workflow schematic for salivary microbiome profiling by 16S rRNA gene sequencing. **(B)** Venn diagram summarizing detected salivary genera in SF and HC, using the detection criterion of relative abundance >0.1% in at least 10% of samples within the corresponding group. **(C)** Differential salivary genera displayed as log2 fold change for SF versus HC; positive values indicate SF enrichment and negative values indicate HC enrichment. Primary FDR-adjusted q values are shown. **(D)** Genus-level row-wise Z-score heatmap across individual salivary samples. **(E, F)** Salivary genus-level composition summarized at group-mean and individual-sample levels. **(G–I)** Phylum-level salivary taxonomic summaries, including detected phyla, differential phyla, and relative-abundance composition. Differential testing used two-sided Mann-Whitney U tests with Benjamini-Hochberg FDR correction within the salivary taxonomic level. The salivary habitat yielded the broadest set of internally stable genus-level candidates after age/sex/BMI adjustment and CLR-based compositional sensitivity analysis. SF, stone formers; HC, healthy controls; CLR, centered log-ratio; FDR, false-discovery rate.

**Figure 5:**
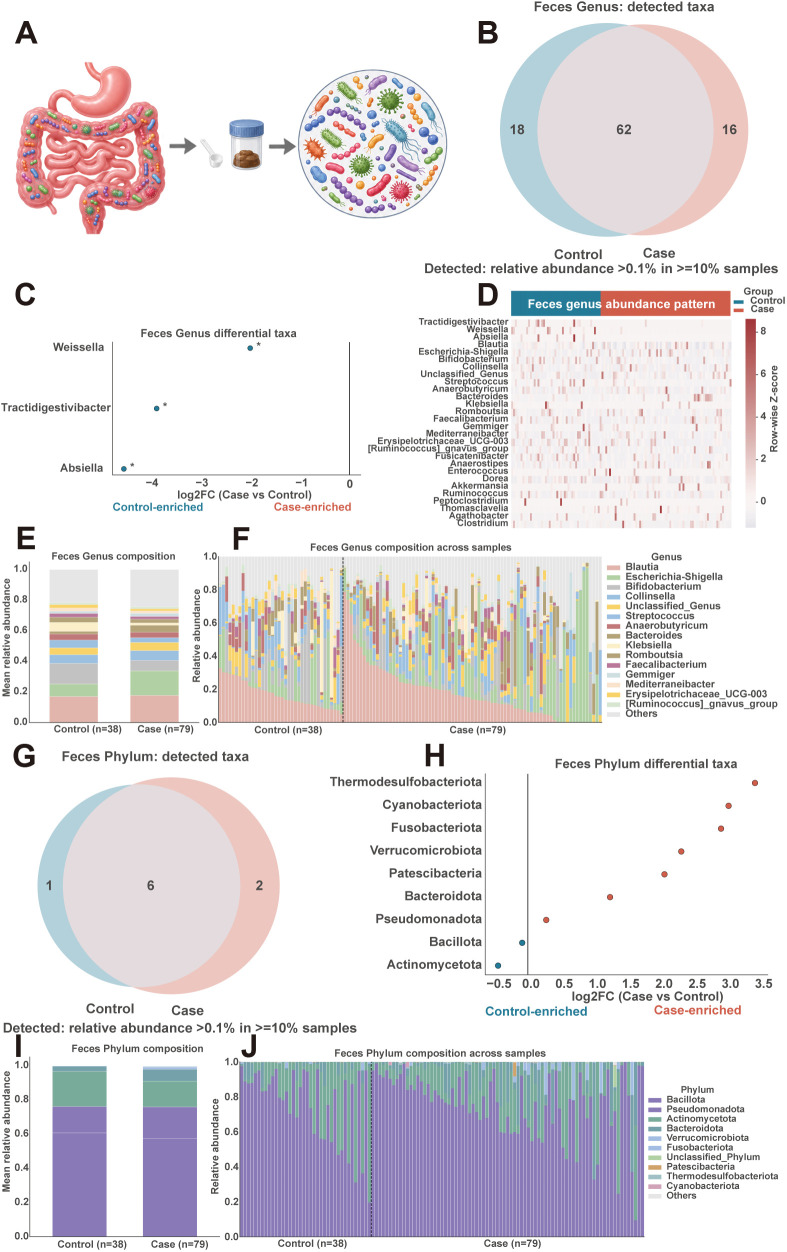
Fecal taxonomic composition and candidate microbial alterations associated with urolithiasis. **(A)** Workflow schematic for fecal microbiome profiling by 16S rRNA gene sequencing. **(B)** Venn diagram summarizing detected fecal genera in SF and HC, using the detection criterion of relative abundance >0.1% in at least 10% of samples within the corresponding group. **(C)** Differential fecal genera displayed as log2 fold change for SF versus HC; positive values indicate SF enrichment and negative values indicate HC enrichment. Primary FDR-adjusted q values are shown. **(D)** Genus-level row-wise Z-score heatmap across individual fecal samples. **(E, F)** Fecal genus-level composition summarized at group-mean and individual-sample levels. **(G–I)** Phylum-level fecal taxonomic summaries, including detected phyla, differential phyla, and relative-abundance composition. Differential testing used two-sided Mann-Whitney U tests with Benjamini-Hochberg FDR correction within the fecal taxonomic level. Fecal taxonomic differences were fewer than those in saliva but were directionally consistent with the reduced richness observed in SF. SF, stone formers; HC, healthy controls; FDR, false-discovery rate.

Taxonomic analysis further resolved the habitat-dependent pattern at both genus and phylum levels (**Figures 3**–**5**). Under the detection criterion of relative abundance >0.1% in at least 10% of samples, prevalence-overlap summaries indicated that SF and HC retained substantial shared taxonomic backbones while also harboring site-specific detectable taxa. At the genus level, urine contained 43 shared genera, with 19 detected only in SF and 25 detected only in HC; saliva contained 66 shared genera, with 10 SF-specific and four HC-specific detected genera; and feces contained 62 shared genera, with 16 SF-specific and 18 HC-specific detected genera. Across the three habitats, group-mean and sample-level abundance profiles showed preservation of the dominant community framework, whereas differences between SF and HC were expressed mainly as finer-scale genus-level redistribution.

In the urinary habitat, 19 genera passed primary FDR-controlled differential testing, comprising six SF-enriched and 13 HC-enriched genera. SF-enriched genera included Finegoldia, Fastidiosipila, Peptostreptococcus, Ruthenibacterium, Hungatella, and Prevotella, whereas HC-enriched genera included Enterococcus, Blautia, [Ruminococcus]_gnavus_group, Fructilactobacillus, Anaerobutyricum, Mediterraneibacter, Fusicatenibacter, Faecalimonas, Eggerthella, CAG-56, Senegalimassilia, Megamonas, and Lachnospiraceae_FCS020_group. After adjustment for age, sex, and BMI, Senegalimassilia and CAG-56 remained adjusted-stable HC-enriched candidates and retained concordant directions in CLR-based composition-aware sensitivity analyses. At the phylum level, eight phyla were detected in both groups and eight additional phyla were detected in SF; however, the mean urinary community in both groups remained dominated by Bacillota, Pseudomonadota, Actinomycetota, and Bacteroidota. Because urine is a low-biomass specimen type and no matched extraction or PCR negative controls were processed in the present study, urinary genus-level associations should be regarded as preliminary candidates pending confirmation in a prospectively collected, negative-control-supported low-biomass workflow.

Saliva exhibited the broadest adjusted-stable genus-level signature. The salivary genus-level overlap profile comprised 66 shared genera, with 10 detected only in SF and four detected only in HC. Primary differential testing identified Neisseria as SF-enriched and 10 genera as HC-enriched. Among the HC-enriched taxa, Mogibacterium, Lancefieldella, Mobiluncus, Solobacterium, Erysipelotrichaceae_UCG-006, [Eubacterium]_sulci_group, Oribacterium, Candidatus_Saccharimonas, and Catonella remained significant with concordant directions after age/sex/BMI adjustment and CLR-based composition-aware sensitivity analysis; Shuttleworthia was observed in the primary analysis but did not fulfil the adjusted-stable definition. At the phylum level, 10 phyla were shared between SF and HC, with one group-specific detected phylum in each group; Pseudomonadota was enriched in SF whereas Patescibacteria was enriched in HC in the displayed differential analysis. These results indicate that the salivary habitat contributed the largest set of internally stable candidate genera distinguishing SF from HC.

Fecal taxonomic differences were fewer at the genus level but were directionally consistent with the fecal richness signal. The fecal overlap profile included 62 shared genera, with 16 detected only in SF and 18 detected only in HC. Three genera, Weissella, Tractidigestivibacter, and Absiella, were HC-enriched in the primary analysis; Tractidigestivibacter and Absiella remained adjusted-stable and directionally concordant in CLR-based sensitivity models. At the phylum level, six phyla were shared, with two detected only in SF and one detected only in HC. As in the other habitats, the overall fecal phylum-level composition was dominated by Bacillota, Pseudomonadota, Actinomycetota, and Bacteroidota despite lower-abundance differential signals.

Together, these results show that taxonomic associations with urolithiasis were not characterized by a single uniform microbial shift across sites. Instead, the three habitats retained broad higher-order community structures while displaying distinct genus-level candidate signatures. Saliva yielded the widest adjusted-stable candidate set, feces showed a smaller set of stable HC-associated genera alongside reduced richness, and urinary genus-level associations remain preliminary pending prospective confirmation with low-biomass negative controls. The full tested-taxa workflow, primary and adjusted differential-abundance results, CLR-based compositional sensitivity analyses, stable-candidate classification, and phylum-level summaries are provided in **Supplementary Figure 6** and **Supplementary Table 4**.

### Multi-habitat profiles provided internally cross-validated discriminatory information beyond a limited clinical baseline

**Figure 6:**
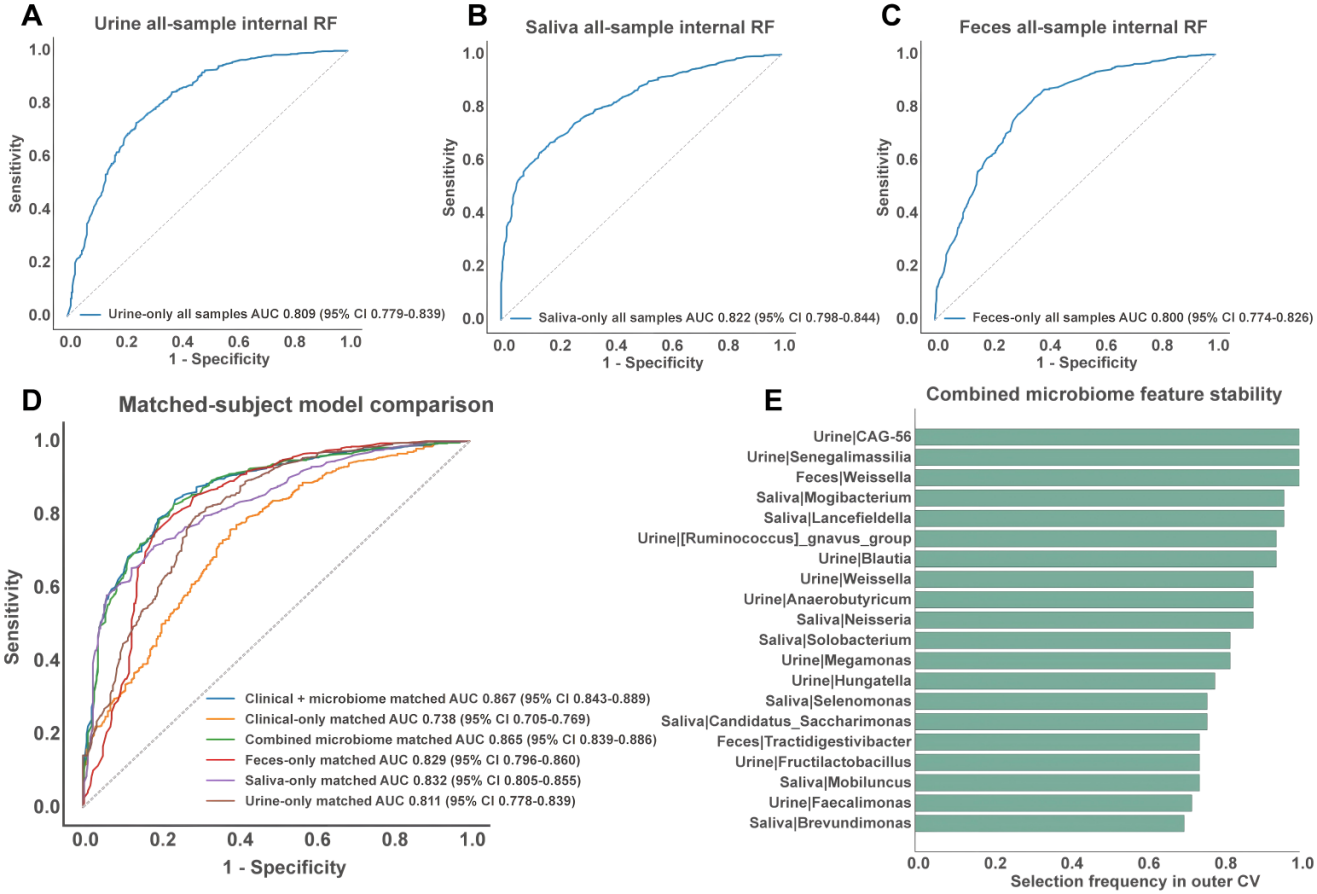
Internal discriminatory performance of random forest models under repeated nested stratified cross-validation. **(A–C)** Receiver operating characteristic (ROC) curves for single-habitat microbiome models fitted in all available samples: **(A)** urine-only, **(B)** saliva-only, and **(C)** feces-only models. **(D)** ROC comparison in the matched three-habitat participant set (n = 98), including urine-only, feces-only, saliva-only, combined multi-habitat microbiome, clinical-only, and clinical-plus-microbiome models. The clinical-only comparator included age, sex, and BMI. **(E)** Selection-frequency ranking of features in the combined multi-habitat microbiome model across outer cross-validation loops. Low-abundance filtering, feature selection, and hyperparameter tuning were performed within the training folds only, and no full-dataset differential-abundance results were used for feature preselection. AUC estimates and 95% confidence intervals summarize internal cross-validated discriminatory information rather than independent diagnostic validation. AUC, area under the receiver operating characteristic curve; CI, confidence interval; ROC, receiver operating characteristic; SF, stone formers; HC, healthy controls.

To evaluate internal discriminatory information, random forest models were assessed using repeated nested stratified cross-validation (**Figure 6**; **Table 2**). Low-abundance filtering, feature selection, and hyperparameter tuning were performed inside training folds only, and no full-dataset differential-abundance results were used for feature preselection within the cross-validation pipeline. This design was used to reduce information leakage and to distinguish exploratory internal discrimination from diagnostic validation.

**Table 2:** Internal random forest model performance.

Model	Analysis set	AUC (95% CI)	Interpretation
Urine-only microbiome	Matched 98 participants	0.811 (0.778-0.839)	Single-habitat internal information
Feces-only microbiome	Matched 98 participants	0.829 (0.796-0.860)	Single-habitat internal information
Saliva-only microbiome	Matched 98 participants	0.832 (0.805-0.855)	Single-habitat internal information
Combined multi-habitat microbiome	Matched 98 participants	0.865 (0.839-0.886)	Numerically higher than the best single-habitat model; paired delta-AUC CI crossed zero
Clinical-only	Matched 98 participants	0.738 (0.705-0.769)	Limited baseline: age, sex, and BMI only
Clinical plus multi-habitat microbiome	Matched 98 participants	0.867 (0.843-0.889)	Higher than the limited clinical-only model in internal cross-validation
Combined microbiome vs best single-habitat microbiome	Subject-level clustered bootstrap	Delta-AUC 0.033 (-0.029 to 0.102)	Difference not statistically conclusive
Combined microbiome vs clinical-only	Subject-level clustered bootstrap	Delta-AUC 0.128 (0.026 to 0.229)	Added internal information beyond the limited age/sex/BMI baseline

In all available habitat-specific samples, urine-only, feces-only, and saliva-only models achieved AUCs of 0.809, 0.800, and 0.822, respectively. In the matched 98-participant three-habitat set, the urine-only model achieved an AUC of 0.811 (95% CI, 0.778-0.839), the feces-only model achieved 0.829 (0.796-0.860), and the saliva-only model achieved 0.832 (0.805-0.855). The combined multi-habitat microbiome model achieved an AUC of 0.865 (0.839-0.886). A clinical-only model based on age, sex, and BMI achieved an AUC of 0.738 (0.705-0.769), whereas a clinical plus multi-habitat microbiome model achieved 0.867 (0.843-0.889).

Subject-level clustered bootstrap comparisons clarified the scale of the added value. The combined microbiome model was numerically higher than the best single-habitat microbiome model, but the delta-AUC of 0.033 had a 95% CI crossing zero (-0.029 to 0.102). The combined microbiome model was higher than the limited clinical-only model based on age, sex, and BMI (delta-AUC 0.128, 95% CI 0.026 to 0.229). Auxiliary subject-averaged metrics were consistent with this internal discriminatory pattern: the combined microbiome and clinical-plus-microbiome models had PR-AUCs of 0.955 and 0.956 and Brier scores of 0.132 and 0.132, respectively, compared with PR-AUC 0.863 and Brier score 0.183 for the limited clinical baseline. The nested cross-validation leakage-control scheme, repeat-level AUC distributions, paired delta-AUC bootstrap comparisons, feature-selection frequencies, PR-AUCs, and Brier score summaries are shown in **Supplementary Figure 7** and **Supplementary Tables 5**, **S6**.

### Exploratory analyses generated habitat-dependent hypotheses

**Figure 7:**
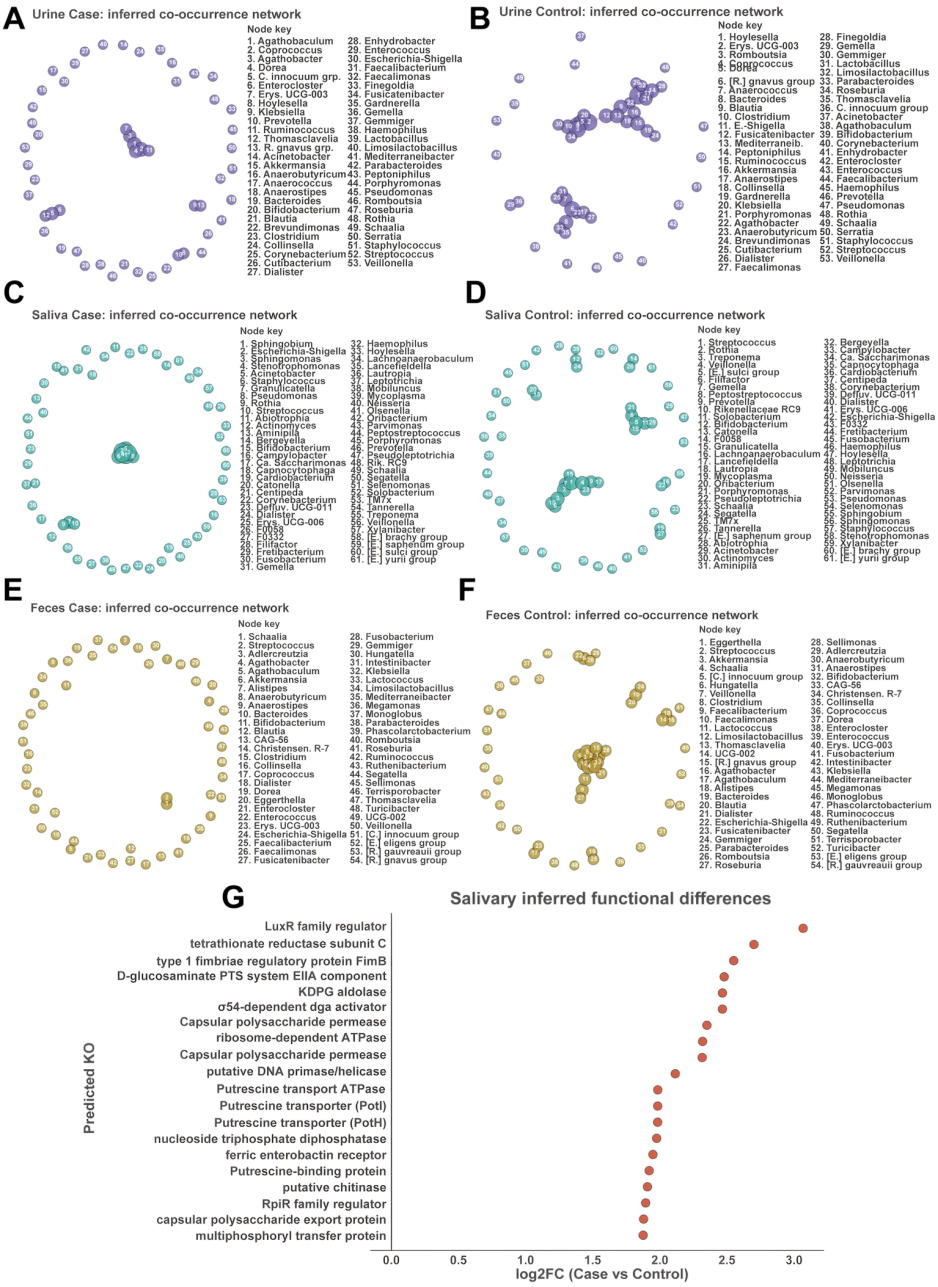
Exploratory inferred co-occurrence network organization and predicted functional potential. **(A, B)** Genus-level inferred co-occurrence networks for urinary microbiota in **(A)** SF and **(B)** HC. **(C, D)** Inferred co-occurrence networks for salivary microbiota in **(C)** SF and **(D)** HC. **(E, F)** Inferred co-occurrence networks for fecal microbiota in **(E)** SF and **(F)** HC. Networks were constructed within each habitat using shared node sets, CLR-transformed genus-level abundance, Spearman correlation thresholds (|rho| > 0.6), Benjamini-Hochberg FDR correction (q < 0.05), and bootstrap edge support (>0.7). Node size is proportional to mean relative abundance. Edges indicate statistically filtered co-occurrence patterns and should not be interpreted as direct microbial interactions. **(G)** Representative salivary KEGG Orthology (KO) features inferred by PICRUSt2 and displayed as log2 fold change for SF versus HC. These KO-level findings represent predicted functional potential rather than directly measured functional activity and require validation using direct functional profiling or targeted assays. CLR, centered log-ratio; FDR, false-discovery rate; HC, healthy controls; KO, KEGG Orthology; SF, stone formers.

Exploratory inferred co-occurrence network analysis suggested habitat-dependent differences in network topology, with the most consistent findings in feces (**Figures 7A–F**). Networks were constructed using shared habitat-specific node sets, CLR-transformed genus-level abundance, Spearman correlation thresholds, FDR correction, bootstrap edge support, and sample-size matched resampling. Observed networks tended to show fewer edges and lower connectivity in cases. After resampling-based empirical FDR correction, fecal differences in edges, density, mean degree, median degree, connected components, and largest component ratio remained significant (q = 0.003). In urine and saliva, only connected components remained significant after empirical FDR correction (q = 0.020). However, these results are interpreted as exploratory topology differences in inferred co-occurrence structure, not as direct microbial interactions. Full sample-size matched resampling distributions and empirical FDR summaries are shown in **Supplementary Figure 8** and **Supplementary Table 7**.

PICRUSt2 analysis was used to summarize predicted functional potential rather than measured function. No primary FDR-significant predicted KEGG Orthology (KO) differences were detected in urinary or fecal samples. In contrast, the salivary habitat showed representative SF-enriched predicted KO signals (**Figure 7G**), including annotations related to bacterial regulation, surface-associated functions, nutrient transport, carbohydrate utilization, and iron acquisition. However, weighted NSTI differed between groups in urine (q = 0.00062) and saliva (q = 0.003), indicating that prediction reliability itself varied by group in these habitats. These KO-level findings were therefore treated as exploratory predicted functional-potential signals requiring validation by shotgun metagenomics, metatranscriptomics, metabolomics, or targeted functional assays. Weighted NSTI distributions, the number of significant KOs by habitat, representative salivary KO effect sizes, and adjusted KO sensitivity summaries are shown in **Supplementary Figure 9** and **Supplementary Table 8**.

**Figure 8:**
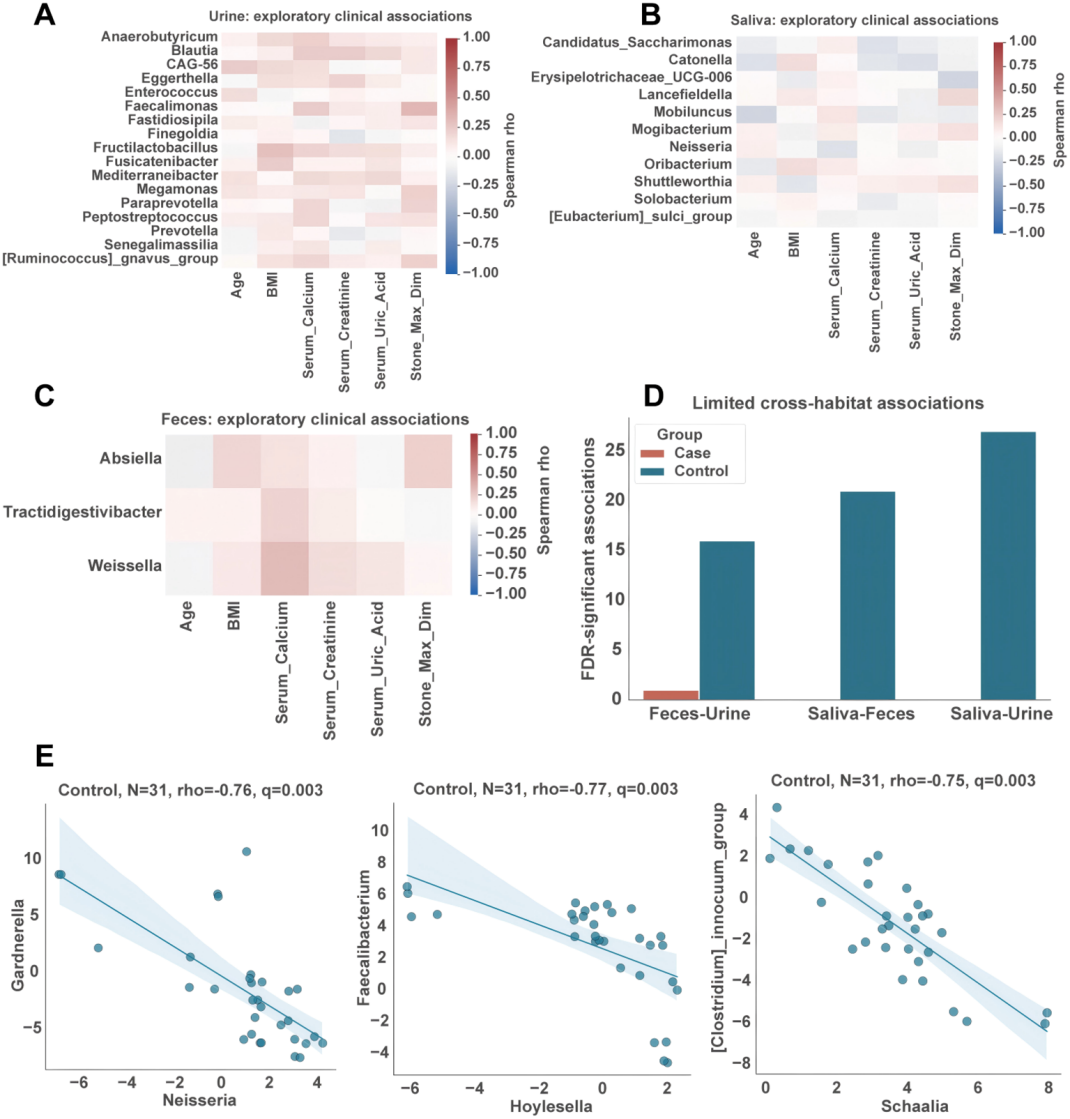
Exploratory clinical-phenotype associations and cross-habitat genus-level covariation. **(A–C)** Spearman correlation heatmaps between candidate discriminatory genera and recorded clinical phenotypes among SF for **(A)** urine, **(B)** saliva, and **(C)** feces. Clinical variables include age, BMI, serum calcium, serum creatinine, serum uric acid, and maximum stone dimension. Color scale indicates Spearman correlation coefficient (rho), and FDR-adjusted q values or significance annotations are shown where applicable. **(D)** Number of FDR-significant cross-habitat genus-level associations detected in SF and HC across feces-urine, saliva-feces, and saliva-urine habitat pairs. **(E–G)** Representative FDR-significant cross-habitat genus correlations in HC: **(E)** urinary Gardnerella versus salivary Neisseria, **(F)** fecal Faecalibacterium versus salivary Hoylesella, and **(G)** fecal [Clostridium] innocuum group versus salivary Schaalia. Shaded areas denote 95% confidence intervals of the fitted linear trend. These exploratory analyses did not support a broad oral-gut-urinary coordination mechanism and should not be interpreted as causal evidence. BMI, body mass index; FDR, false-discovery rate; HC, healthy controls; SF, stone formers.

Clinical-phenotype, stone-composition, and cross-habitat analyses were conducted as exploratory extensions of the primary microbiome findings. No taxon-clinical phenotype association passed FDR correction within any habitat. Stone-composition data were available for all 80 SF in the standardized metadata [calcium oxalate (CaOx), n = 58; calcium phosphate (CaP), n = 14; uric acid (UA), n = 8], but subgroup analyses were underpowered and did not support subtype-specific microbiome conclusions. Exploratory clinical-correlation heatmaps are shown in **Figures 8A–C**, with stone-composition subgroup sizes and subgroup test summaries provided in **Supplementary Figure 10** and **Supplementary Table 9**. Cross-habitat genus-level covariation was sparse after FDR correction and did not reveal a consistent SF-specific covariation pattern. These results did not support a broad oral-gut-urinary coordination mechanism. Tested-pair counts and FDR-significant pair-count summaries are shown in **Supplementary Figure 11** and **Supplementary Table 9**, with FDR-significant cross-habitat association counts and representative associations additionally shown in **Figures 8D–G**; the external evidence-boundary summary is provided in **Supplementary Figure 12** and **Supplementary Table 12**.

### External contextual analyses provided partial urinary support and oral epidemiological context

External contextual analyses were performed using publicly available KiSMi and NHANES datasets (**Supplementary Figures 1**, **S2**, **S12**; **Supplementary Tables 10**–**S12**). Reconciled KiSMi processed matrices provided partial support for urinary community-level alteration, whereas salivary community-level findings and prespecified genus-level candidates were not consistently reproduced after harmonization. The KiSMi gut shotgun comparison was retained as a cross-platform exploratory analysis rather than a direct fecal 16S replication. In NHANES 2009–2012 oral-rinse data, observed oral ASV richness showed an inverse association with self-reported kidney-stone history after adjustment for measured dietary, water-intake, socioeconomic, clinical, and demographic covariates, although this association was FDR-non-significant in the fully adjusted population-based model. Together, these public-data analyses provided external context for selected habitat-specific signals, particularly urinary community-level alteration, while indicating that direct replication will require harmonized specimen collection, sequencing strategy, covariate capture, and outcome definition.

## Discussion

By simultaneously profiling urinary, fecal, and salivary microbial communities in the same individuals, this study delineates a multi-habitat microbiome landscape associated with urolithiasis. Three findings merit emphasis. First, SF and HC showed statistically detectable differences in microbial community structure across all three sampled habitats, indicating that urolithiasis is accompanied by microbial alterations beyond a single anatomical site. Second, the form of these alterations differed by habitat: feces showed reduced richness, whereas saliva and urine contributed distinct genus-level candidate signatures without broad alpha-diversity loss. Third, integrated multi-habitat microbial profiles provided additional internally cross-validated discriminatory information beyond a limited clinical baseline consisting of age, sex, and BMI. Collectively, these findings support a habitat-dependent model of microbiome alteration in urolithiasis and establish simultaneous multi-site sampling as a useful strategy for capturing complementary disease-associated microbial information.

The fecal findings provide an important ecological dimension to the microbiome changes associated with stone disease. The selective reduction in richness indices (Sobs, Chao1, and ACE), together with preserved evenness and no uniform diversity loss across all habitats, suggests that stone-associated intestinal dysbiosis may involve depletion of selected low-abundance taxa rather than wholesale community collapse. This pattern is biologically relevant because intestinal microbes have been implicated in oxalate handling, short-chain fatty acid production, bile-acid metabolism, epithelial barrier regulation, and systemic inflammatory homeostasis (Ticinesi et al., 2018; Kim et al., 2022; Hunthai et al., 2024; An et al., 2025; Jia et al., 2025; Li D. et al., 2025; Liu L. et al., 2025; Chen et al., 2026; Cui et al., 2026). The fecal richness reduction observed here therefore complements previous mechanistic and observational evidence linking intestinal microbial ecology to stone disease, while also providing a concrete habitat-specific signal for longitudinal validation.

The urinary and salivary findings further demonstrate that disease-associated microbiome information is not restricted to broad diversity loss. Although neither habitat showed FDR-significant alpha-diversity differences, both displayed case-control community-distribution differences and habitat-specific taxonomic redistribution. Saliva contributed the broadest set of covariate-robust genus-level candidates, highlighting the potential value of this readily accessible niche for repeatable, minimally invasive microbial profiling. In urine, genus-level candidates are biologically attractive because the urinary habitat is anatomically closest to crystals, urinary mucosa, local inflammatory conditions, and stone-associated microenvironments. These candidates therefore provide focused targets for future low-biomass studies that incorporate extraction blanks, PCR controls, quantitative microbial-biomass assessment, and contamination-aware statistical workflows.

The machine-learning analysis adds a translational dimension to the multi-habitat design. Under repeated nested cross-validation, the combined multi-habitat microbiome model achieved an AUC of 0.865 and exceeded the limited age/sex/BMI clinical baseline by a delta-AUC of 0.128, with a 95% CI excluding zero. This result supports the presence of disease-associated microbial information beyond basic demographic and anthropometric variables. The combined microbiome model was not conclusively superior to the best single-habitat model, which appropriately limits diagnostic claims; nevertheless, the findings indicate that microbial features collected across different habitats contain clinically relevant, partly complementary information that warrants prospective evaluation alongside urine chemistry, dietary exposure, fluid intake, stone composition, recurrence history, and treatment outcomes (Liu Y. et al., 2025; Zhang M. et al., 2025).

The exploratory network and predicted-functional analyses offer additional biological directions arising from the primary observations. Inferred network topology differences were most consistent in feces, where SF showed reduced connectivity across several sample-size matched, resampling-supported metrics. When considered together with reduced fecal richness, this pattern suggests that the intestinal microbiome associated with urolithiasis may exhibit not only a narrower taxonomic repertoire but also altered ecological organization. In saliva, PICRUSt2 identified SF-enriched predicted KO annotations related to bacterial regulation, surface-associated functions, nutrient transport, carbohydrate utilization, and iron acquisition. Although 16S-based functional prediction cannot substitute for direct functional measurement (Matchado et al., 2024), these results provide specific hypotheses for shotgun metagenomic, metatranscriptomic, metabolomic, or targeted functional studies.

A conceptual contribution of this study is that it separates the value of multi-habitat sampling from the requirement for a single coordinated oral-gut-urinary dysbiosis mechanism. The observed microbial alterations were informative within habitats, whereas cross-habitat genus-level covariation was sparse after FDR correction. This finding does not diminish the importance of multi-site profiling. Instead, it suggests that different microbial habitats may contribute complementary information because they capture distinct local ecological responses associated with urolithiasis: the fecal microbiome may reflect intestinal ecological breadth and metabolic context, the urinary microbiome may capture locally situated candidates proximal to the stone environment, and the salivary microbiome may provide accessible discriminatory features with translational potential. Accordingly, the present study advances a refined framework in which urolithiasis-associated microbiome alterations are habitat dependent, complementary, and potentially integrable for clinical investigation.

The present findings extend and partially refine prior multi-site microbiome work in kidney stone disease. Al et al. (2023) reported multi-site microbiota alteration as a hallmark of stone formation using 16S rRNA gene sequencing of fecal, salivary, and urinary samples. Our study confirms that stone-associated microbial alterations are detectable across multiple body sites, while adding a larger internal cohort with 98 matched three-habitat profiles, repeated nested cross-validation, covariate-adjusted taxonomic sensitivity analyses, and public-dataset contextual evaluation. Compared with a broad cross-site dysbiosis interpretation, our results support a more site-resolved view: multi-habitat profiling is informative not because all sites necessarily move together, but because each niche contributes a distinct layer of disease-associated microbial information.

The external contextual analyses further help position these findings for future validation. KiSMi processed matrices provided partial community-level support for urinary microbial alteration but did not consistently reproduce salivary community-level or prespecified genus-level findings, likely reflecting differences in specimen type, platform, filtering strategy, participant composition, and available covariates. NHANES 2009–2012 oral-rinse analysis enabled a population-based sensitivity evaluation under broader adjustment for demographic, dietary, water-intake, socioeconomic, and clinical variables; the inverse association between observed oral ASV richness and self-reported kidney-stone history did not remain FDR-significant in the fully adjusted model. These public datasets are therefore best viewed as external context rather than direct validation cohorts, but they clarify feasible directions for harmonized prospective replication.

Several limitations should be acknowledged. The cross-sectional design does not permit causal inference, and differences in age and sex between SF and HC, together with unavailable internal data on detailed diet, habitual fluid intake, selected medication exposures, socioeconomic status, urine chemistry, and recurrence phenotype, leave residual confounding possible despite sensitivity analyses. Urinary genus-level findings require particular caution because urine is a low-biomass specimen and matched negative controls were not available in the retrospective workflow. Sample attrition differed across habitats, functional analyses were predicted from 16S profiles rather than directly measured, and the internally cross-validated models require independent prospective validation. These limitations define the next priorities: longitudinal multi-habitat sampling, rigorous low-biomass control workflows, richer clinical and exposure metadata, direct functional profiling, and prespecified external validation.

## Conclusion

In conclusion, this unified urinary, fecal, and salivary 16S rRNA gene sequencing study identifies habitat-dependent microbiome alterations associated with urolithiasis. The three habitats contributed distinct but complementary signals: reduced fecal richness, the broadest covariate-robust taxonomic candidate set in saliva, and biologically proximal urinary genus-level candidates that require contamination-controlled validation. Integrated multi-habitat microbial profiles provided internally cross-validated discriminatory information beyond a limited clinical baseline of age, sex, and BMI. These findings support simultaneous multi-site profiling as a valuable framework for understanding urolithiasis-associated microbial ecology and for developing future biomarker and mechanistic studies. Prospective investigations incorporating rigorous low-biomass controls, comprehensive exposure and urine-chemistry metadata, direct functional measurements, recurrence outcomes, and prespecified external validation are warranted to confirm and extend these site-specific and integrative microbial signatures.

## Methods and materials

### Study design, participants, and sample collection

This study was approved by the Ethics Committee of the First Affiliated Hospital of Sun Yat-sen University (ethics approval No [2024]. 757), and written informed consent was obtained from all participants before enrollment. A total of 80 patients with urolithiasis were enrolled as SF, and 40 healthy individuals were recruited as HC. Eligible SF were adults aged 18 years or older with a clinical diagnosis of upper urinary tract calculi. Diagnosis was based on routine clinical imaging, including urinary ultrasonography, abdominal computed tomography, or other standard radiological examinations indicating the presence of stones, and was confirmed by medical-record review.

Exclusion criteria were use of antibiotics, immunosuppressants, or corticosteroids within the preceding 3 months; severe hepatic or renal dysfunction, malignancy, or other major systemic diseases; gastrointestinal diseases or functional disorders, including irritable bowel syndrome, chronic diarrhea, or constipation (Shen et al., 2024); smoking; and, in women, menstruation, pregnancy, or lactation. Healthy controls had no history of urolithiasis and had normal findings on contemporaneous physical examination or urinary imaging assessment. Under fasting conditions, saliva, clean-catch midstream urine, and fresh fecal samples were collected using aseptic procedures. Samples were transported under low-temperature conditions immediately after collection and stored at -80 °C until further processing.

Internal analyses used the standardized ASV count table, ASV-level taxonomy annotation, metadata workbook, and sample-renaming table generated for the final analysis. Sample IDs, subject IDs, habitat labels, and group labels were harmonized before analysis. Of 120 enrolled participants, salivary records were available for all 80 SF and 40 HC; fecal records were available for 79 SF and 40 HC, of which 79 SF and 38 HC had matching ASV count columns; urinary records were available for 68 SF and 40 HC, of which 68 SF and 33 HC had matching ASV count columns. Thus, the final internal analytic dataset contained 101 urinary samples, 117 fecal samples, and 120 salivary samples, with 98 participants contributing all three habitats. Detailed pre-analytical reasons for absent urinary or fecal records were not fully recoverable retrospectively and may include noncollection, insufficient material, amplification/sequencing failure, or upstream quality-control exclusion; this habitat-specific missingness was treated as a potential source of selection bias. Core covariates for sensitivity analyses were age, sex, and BMI. Diet, habitual fluid intake, NSAID use, antispasmodic use, other non-antibiotic medication exposure, and socioeconomic variables were not available as reliable analyzable internal fields. Sample retention, rarefaction eligibility, and the sample-level exclusion log are summarized in **Supplementary Figure 4** and **Supplementary Table 1**.

### PCR amplification, library construction, and high-throughput sequencing

Total DNA extracted from each specimen was used as the template for amplification of the bacterial 16S rRNA gene. No extraction blanks or PCR negative controls were processed for this retrospective sample collection, and formal contaminant identification or decontamination modeling could therefore not be performed. Total genomic DNA of the microbial community was extracted according to the manufacturer’s instructions of the PF Mag-Bind Stool DNA Kit (Omega Bio-tek, Georgia, U.S.). The integrity of the extracted genomic DNA was assessed by 1% agarose gel electrophoresis, and DNA concentration and purity were measured using a NanoDrop 2000 spectrophotometer (Thermo Scientific, U.S.). The V3-V4 hypervariable region was amplified using universal primers 338F (5’-ACTCCTACGGGAGGCAGCAG-3’) and 806R (5’-GGACTACHVGGGTWTCTAAT-3’), each containing a sample-specific barcode sequence. PCR amplification was performed in a total reaction volume of 20 microliters, containing 4 microliters of 5x TransStart FastPfu buffer, 2 microliters of 2.5 mM dNTPs, 0.8 microliters of forward primer (5 micromolar), 0.8 microliters of reverse primer (5 micromolar), 0.4 microliters of TransStart FastPfu DNA polymerase, 10 ng of template DNA, and sterile ddH2O to the final volume. Thermocycling conditions were initial denaturation at 95 degrees C for 3 min; 27 cycles of denaturation at 95 degrees C for 30 s, annealing at 55 degrees C for 30 s, and extension at 72 degrees C for 30 s; final extension at 72 degrees C for 10 min; and holding at 4 degrees C. Amplification was performed on an ABI GeneAmp 9700 thermocycler.

Amplicons were examined by 2% agarose gel electrophoresis, excised from the gel, and purified using a DNA gel extraction kit. Purified PCR products were quantified using a Qubit 4.0 fluorometer. Sequencing libraries were constructed using the NEXTFLEX Rapid DNA-Seq Kit, including adapter ligation, bead-based size selection, PCR enrichment, and a second bead-purification step. Qualified libraries were subjected to paired-end sequencing on the Illumina NextSeq 2000 platform by Majorbio Bio-Pharm Technology Co., Ltd. (Shanghai, China).

### Quality control, ASV construction, and taxonomic annotation

Raw paired-end sequencing reads were quality-filtered using fastp (version 0.23.4) and merged using FLASH (version 1.2.11). Bases with quality scores below 20 were trimmed from the 3’ end, and a 50-bp sliding window was applied; when the average quality score within a window dropped below 20, reads were truncated at that position. Reads shorter than 50 bp after filtering and reads containing more than five ambiguous bases were discarded.

Paired-end reads passing quality filtering were merged according to overlap relationships, using a minimum overlap length of 10 bp and a maximum mismatch ratio of 0.2 within the overlapping region. Samples were distinguished and sequence orientation was standardized on the basis of barcode and primer information at both ends. No barcode mismatches were allowed, and up to two primer mismatches were permitted.

High-quality merged sequences were denoised in QIIME2 using the DADA2 plugin with default parameters to generate amplicon sequence variants (ASVs). Sequences annotated as chloroplasts or mitochondria were removed. Taxonomic annotation was performed in QIIME2 using the classify-sklearn Naive Bayes classifier against the SILVA 138.2 16S rRNA gene reference database, with the confidence threshold set at 70%. The ASV-level abundance matrix contained 21,983 ASVs before downstream rarefaction. Samples with fewer than 500 high-quality merged reads were excluded during initial sequencing-quality screening. For alpha- and beta-diversity analyses requiring rarefaction, only samples with at least 22,254 reads were retained and rarefied to 22,254 reads per sample using random seed 42. Taxonomic relative-abundance analyses, random forest modeling, inferred-network analysis, and PICRUSt2-based predicted functional-potential analysis used the final sample set after sample reconciliation and feature filtering as specified below; each analysis table reports the actual habitat-specific sample size.

### Alpha- and beta-diversity analyses

Rarefied ASV tables were used to calculate Sobs, Chao1, ACE, Shannon, Simpson, Pielou’s evenness, and Good’s coverage. Group comparisons were performed separately within each habitat using two-sided Mann-Whitney U tests, followed by Benjamini-Hochberg FDR correction within each habitat and a global 21-comparison sensitivity correction. Core-adjusted sensitivity models used rank-transformed alpha-diversity metrics with group, age, sex, and BMI as covariates. Full alpha-diversity sensitivity outputs, including male-restricted results, are provided in **Supplementary Figure 5** and **Supplementary Table 2**.

Beta-diversity was analyzed using Bray-Curtis and Jaccard distance matrices. Principal coordinates analysis was used for visualization, PERMANOVA was used to test group effects, and PERMDISP was used to assess differences in within-group dispersion. Because PERMDISP was significant, distance-to-centroid summaries were additionally generated to visualize dispersion differences. PERMANOVA results were interpreted as community-distribution differences rather than complete group separation. Distance-to-centroid summaries and the full PERMANOVA/PERMDISP table are provided in **Supplementary Figure 3** and **Supplementary Table 3**.

### Taxonomic composition and differential taxa

ASV count tables were aggregated to phylum and genus levels using ASV-level taxonomy annotation. Composition plots, prevalence-overlap summaries, and heatmaps were used descriptively. Detection in overlap summaries required relative abundance >0.1% in at least 10% of samples within the corresponding group. Primary differential taxon testing used fixed feature filtering, two-sided Mann-Whitney U tests, and BH-FDR correction within each site and level. Core-adjusted sensitivity models were run for all filtered taxa using rank-transformed relative abundance and age, sex, and BMI. Adjusted-stable candidates required primary q <0.05, adjusted-model q <0.05, and concordant direction. A CLR-based composition-aware sensitivity model with age, sex, and BMI was added for genus-level candidates emphasized in the main text. Throughout this study, genera meeting all three of the following criteria were designated as covariate-robust (termed “adjusted-stable” in supplementary tables): (i) primary FDR q < 0.05, (ii) age/sex/BMI-adjusted model FDR q < 0.05, and (iii) concordant direction of effect between primary and adjusted analyses. The tested-taxa workflow, CLR sensitivity results, and stable-candidate classification are summarized in **Supplementary Figure 6** and **Supplementary Table 4**.

### Candidate discriminatory features and random forest modeling

Candidate discriminatory genera were summarized using effect-size plots. Random forest models were evaluated using repeated nested stratified cross-validation. The outer loop used 5-fold cross-validation repeated 10 times for performance estimation, and the inner loop used 5-fold cross-validation for tuning and feature selection. Low-abundance filtering, feature selection, and parameter selection were performed inside each training fold only. No differential-abundance result computed using the full dataset was used for feature preselection within the random forest cross-validation pipeline. Class imbalance was addressed using balanced class weights.

Single-habitat models were fit for urine, feces, and saliva. Matched analyses were restricted to participants with all three habitats available. For the combined microbiome model, genus-level feature matrices from the three habitats were horizontally concatenated with habitat prefixes. A limited clinical-only model included age, sex, and BMI. A clinical-plus-microbiome model combined these clinical variables with multi-habitat microbiome features. Primary AUC estimates were derived from repeated outer-fold predictions; subject-level clustered bootstrap was used for paired delta-AUC comparisons, and auxiliary subject-averaged PR-AUC, Brier score, sensitivity, specificity, and balanced accuracy summaries were generated to describe model behavior. Model-stability plots and feature-selection summaries are provided in **Supplementary Figure 7** and **Supplementary Tables 5**–**S6**.

### Exploratory inferred network analysis

Inferred network analysis was exploratory and was based on genus-level relative abundance. Within each habitat, genera were retained if they had prevalence >=20% and mean relative abundance >=0.1% across all samples. The same node set was used for cases and controls within each habitat. Abundances were transformed using centered log-ratio transformation after adding a pseudocount. Edges were defined by Spearman correlations with absolute rho >0.6, Benjamini-Hochberg FDR q <0.05, and bootstrap support >0.7. Topological metrics included node number, edge number, density, mean and median degree, clustering coefficient, connected components, largest component ratio, modularity, and positive-edge proportion. To address case-control sample-size imbalance, 1000 sample-size matched resamplings were performed, and empirical FDR correction was applied. Sample-size matched topology sensitivity analyses are shown in **Supplementary Figure 8** and **Supplementary Table 7**.

### PICRUSt2 predicted functional-potential analysis

Predicted functional potential was assessed using ASV x KEGG Orthology prediction matrices generated by PICRUSt2. These matrices were multiplied by the final ASV sample count tables to generate sample-level predicted KO abundance profiles. Primary comparisons used Mann-Whitney U tests within each habitat with Benjamini-Hochberg FDR correction across tested KOs. Core-adjusted rank-based models including age, sex, and BMI were run for tested KOs where estimable. Weighted NSTI values were summarized and compared between groups to evaluate the reliability of 16S-based functional prediction. PICRUSt2 results were interpreted as predicted functional potential, not directly measured function, with particular caution for urinary samples because urine is a low-biomass habitat and because group differences in weighted NSTI can affect prediction reliability. Weighted NSTI distributions and predicted-KO sensitivity summaries are shown in **Supplementary Figure 9** and **Supplementary Table 8**.

### Clinical phenotype, stone composition, and cross-habitat association analyses

Clinical phenotype analyses used the covariate-robust candidate discriminatory genera described above and were restricted to SF. Continuous clinical variables included age, BMI, serum calcium, serum uric acid, serum creatinine, and maximum stone dimension. Spearman correlations were calculated where sufficient valid samples were available, and p values were adjusted using Benjamini–Hochberg FDR correction within each habitat. Core-adjusted rank models were fit for prespecified taxon–phenotype pairs where estimable. These analyses were exploratory; no taxon–clinical phenotype association passed FDR correction within any habitat in the primary analysis. Clinical-correlation and stone-composition exploratory results are summarized in **Supplementary Figure 10** and **Supplementary Table 9**.

Stone-composition subgroup analysis was also restricted to SF. Standardized categories in the final clinical metadata were calcium oxalate (CaOx, n = 58), calcium phosphate (CaP, n = 14), and uric acid (UA, n = 8). Given the limited sample sizes of the CaP and UA subgroups, stone composition analyses were restricted to Kruskal–Wallis screening and interpreted as hypothesis-generating. Kruskal–Wallis tests were used only when at least three composition subgroups had n ≥ 5; *post hoc* Dunn tests were considered only after a significant overall test. These analyses were exploratory and were not used to infer composition-specific microbiome mechanisms.

Cross-habitat association analysis used the same 98 matched participants as the combined random forest analysis. Each habitat was filtered at prevalence >=20% and mean relative abundance >=0.1%, transformed using CLR after pseudocount addition, and analyzed with Spearman correlations for saliva-urine, saliva-feces, and feces-urine genus pairs. FDR correction was performed across tested cross-habitat pairs within case and control strata. The analysis was intended to describe cross-site covariation and was not interpreted as a causal or mechanistic axis analysis. Tested-pair counts and FDR-significant sparse association summaries are shown in **Supplementary Figure 11** and **Supplementary Table 9**.

### KiSMi external community and genus-level contextual analysis

Public KiSMi processed matrices were analyzed only as external contextual analyses. Because specimen type, platform, feature filtering, participant composition, and covariate structure differed from the internal cohort, they were not treated as direct replication cohorts. Full reconciliation steps, model specifications, evidence-boundary summaries, and complete results are provided in **Supplementary Figure 1** and **S12** and **Supplementary Table 10** and S12.

For KiSMi, reconciled urinary, salivary, and gut shotgun processed matrices were used without FASTQ reprocessing. Urine and saliva community-level analyses used zero replacement, CLR transformation, Aitchison distance, PCA visualization, PERMANOVA, and PERMDISP. Prespecified internal genus candidates were harmonized to KiSMi genus labels when possible, and gut shotgun comparisons were treated as cross-platform exploratory analyses only.

### NHANES external population-based oral microbiome sensitivity analysis

For NHANES, publicly available 2009–2012 oral-rinse microbiome alpha-diversity data were analyzed as an external population-based epidemiological sensitivity analysis. The outcome was self-reported history of kidney stones, and the primary exposure was observed ASVs at 10,000 reads, averaged across 10 rarefactions. Survey-weighted logistic models incorporated the NHANES design variables and appropriate four-year weights; fully adjusted models included demographic, clinical, socioeconomic, plain-water intake, and dietary covariates. NHANES workflow and model summaries are provided in **Supplementary Figure 2** and **Supplementary Table 11**.

These external analyses were used to define interpretive boundaries. They cannot validate salivary findings directly, cannot evaluate urinary or fecal confounding in the internal cohort, and cannot support cross-habitat coordination or causality; this evidence boundary is summarized in **Supplementary Figure 12** and **Supplementary Table 12**.

### Statistical analysis and reporting

Unless otherwise specified, statistical tests were two-sided, and FDR-adjusted q values were used to determine statistical significance in the main text. Raw p values, within-family q values, and global sensitivity q values were reported in **Supplementary Tables** with explicit labels. Internal analyses were performed primarily in Python using pandas, NumPy, SciPy, scikit-bio, statsmodels, scikit-learn, NetworkX, matplotlib, and related packages. NHANES survey-weighted analyses used SDMVPSU, SDMVSTRA, and appropriate four-year weights, with Day-1 dietary weights for models containing water or dietary variables. Analyses with limited sample size, low-biomass uncertainty, predicted-function inference, cross-sectional design, or lack of independent prospective validation were interpreted as exploratory.

## Data Availability

The raw 16S rRNA gene sequencing reads generated in this study have been deposited in the NCBI Sequence Read Archive under BioProject accession number PRJNA1479366. The deposited data include the raw paired-end sequencing files for urinary, fecal, and salivary samples analyzed in this study, with sample identifiers de-identified before submission. No directly identifiable participant information has been included in the public archive. These raw sequence data constitute the primary publicly available dataset for this study and enable independent reprocessing of the microbiome sequencing data from the original reads. Access to additional clinical information is restricted because of participant privacy, institutional ethical requirements, and applicable data-protection regulations. Reasonable requests for further clarification regarding the deposited sequencing dataset may be directed to the corresponding author.

## Supplementary material

Supplementary Table 1 Sample availability, quality-control retention, sequencing-depth summary, metadata missingness, and sample inclusion/exclusion log by habitat and group.

Supplementary Table 2 Alpha-diversity primary tests, global FDR sensitivity results, age/sex/BMI-adjusted rank-based models, and male-restricted sensitivity analysis.

Supplementary Table 3 PERMANOVA, PERMDISP, core-adjusted beta-diversity models, distance-to-centroid values, and centroid-summary statistics.

Supplementary Table 4 Differential taxa by habitat and taxonomic level, including primary tests, core-adjusted models, CLR-based composition-aware sensitivity analyses, and stable-candidate classification.

Supplementary Table 5 Random forest model-performance summary, including AUC, confidence intervals, paired delta-AUC, PR-AUC, Brier score, sensitivity, specificity, and balanced accuracy.

Supplementary Table 6 Random forest feature-selection frequency and mean feature-importance summaries across nested cross-validation folds.

Supplementary Table 7 Exploratory inferred-network observed topology metrics and sample-size matched resampling summaries with empirical q values.

Supplementary Table 8 PICRUSt2 predicted KO statistics, age/sex/BMI-adjusted KO sensitivity models, sample-level weighted NSTI values, and NSTI group comparisons.

Supplementary Table 9 Exploratory clinical-phenotype correlations, stone-composition subgroup counts and tests, and cross-habitat association summaries.

Supplementary Table 10 KiSMi external contextual analysis outputs, including community-level statistics, PERMDISP results, prespecified genus-level replication categories, and gut shotgun exploratory comparisons.

Supplementary Table 11 NHANES oral-rinse epidemiological sensitivity analysis outputs, including cohort flow, survey-weighted alpha-diversity models, sensitivity analyses, and missingness summaries.

Supplementary Table 12 Internal-external taxonomy harmonization, feature-level compatibility, replication-category summary, and external evidence-boundary documentation.
