# Supplementary material for: Multi-habitat microbiome profiling identifies habitat-dependent alterations and complementary discriminatory information in urolithiasis
Source: Front Cell Infect Microbiol. 2026 Jul 16;16:1853826. doi: 10.3389/fcimb.2026.1853826 (PMC13422501; doi:10.3389/fcimb.2026.1853826)
Supplement: Supplementary file 2 [file DataSheet2.docx]

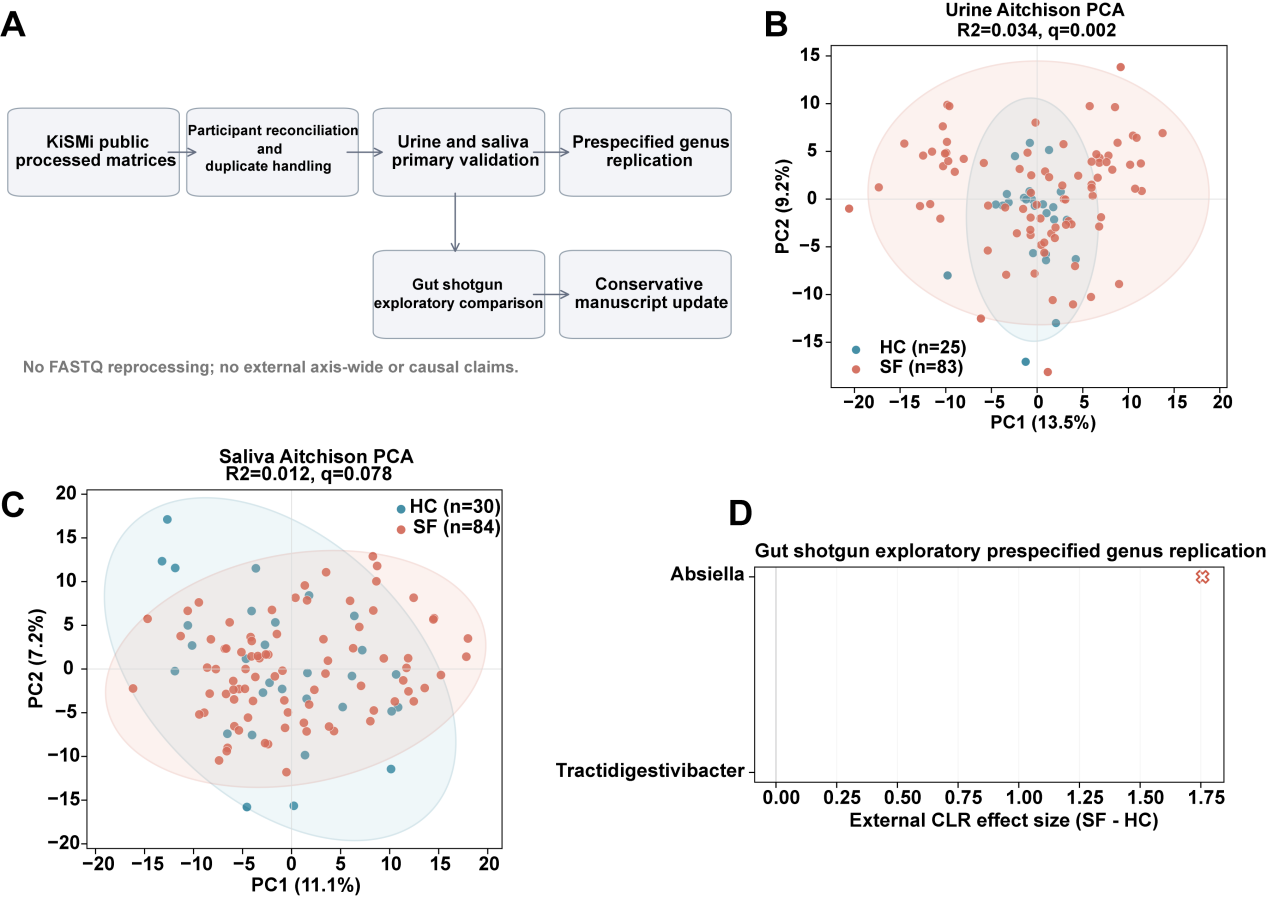


**Supplementary Figure S1. External contextual analysis in the public KiSMi cohort.** (A) Workflow for processed-matrix selection, participant reconciliation, duplicate-sample handling, taxonomy harmonization, and separation of urinary/salivary contextual analyses from exploratory gut shotgun comparisons. (B) CLR-Aitchison principal-component analysis of the reconciled KiSMi urinary processed sequence-variant matrix comparing SF with HC. The displayed PERMANOVA R2 and FDR-adjusted q value summarize the group-associated community-level signal; dispersion diagnostics are reported in the accompanying supplementary table. (C) CLR-Aitchison principal-component analysis of the reconciled KiSMi salivary processed sequence-variant matrix using the same compositional framework as in panel B. (D) Exploratory gut shotgun genus-level comparison for internally prespecified fecal candidate genera. KiSMi analyses were used as external contextual evidence and were not treated as direct replication of the internal 16S multi-habitat findings. CLR, centered log-ratio; FDR, false-discovery rate; HC, healthy controls; SF, stone formers.


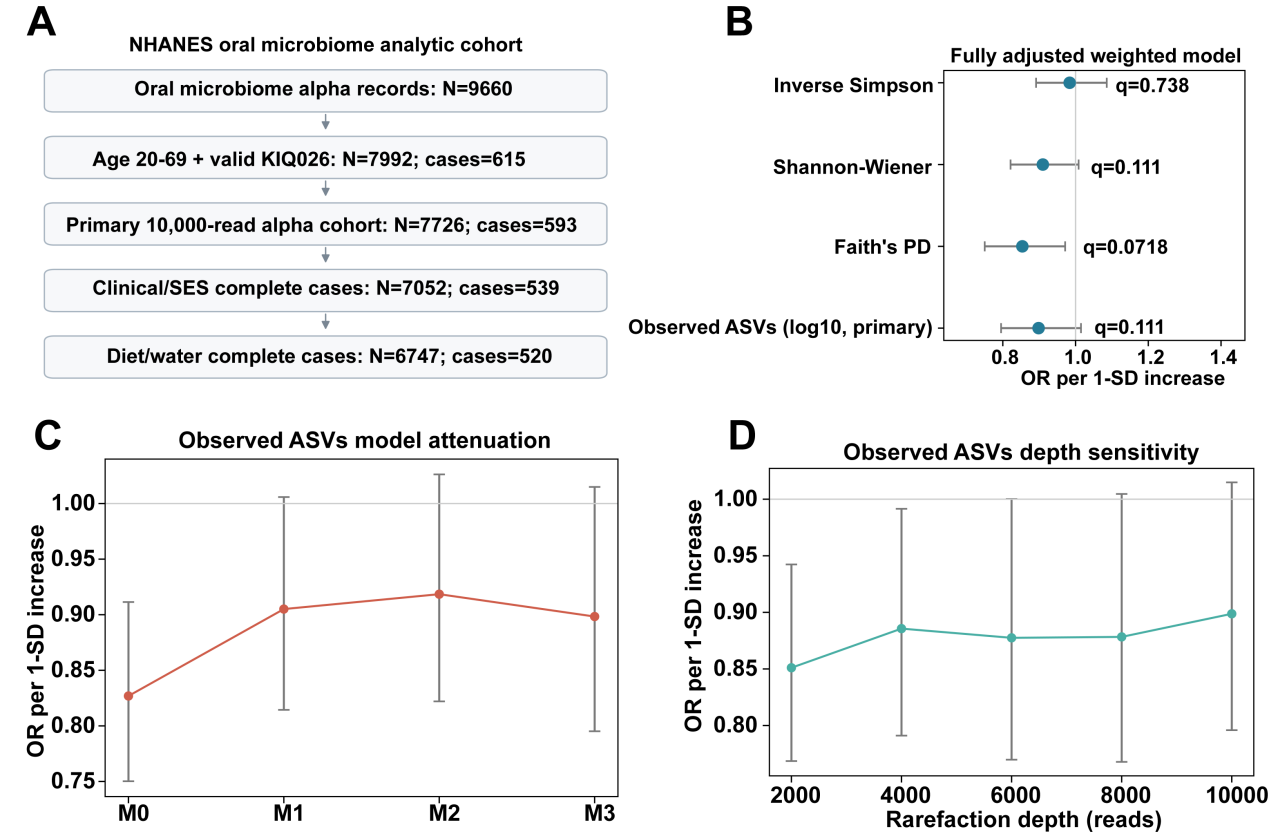


**Supplementary Figure S2. NHANES population-based oral microbiome epidemiological sensitivity analysis.** (A) Analytic cohort selection from eligible NHANES oral microbiome records to adults aged 20-69 years with valid self-reported kidney-stone history, 10,000-read alpha-diversity data, and complete covariate information for the fully adjusted dietary and water-intake model. (B) Survey-weighted fully adjusted logistic regression results for oral alpha-diversity metrics. Odds ratios are reported per 1-SD increase in each metric, with 95% confidence intervals and Benjamini-Hochberg FDR-adjusted q values across the alpha-diversity testing family. (C) Sequential adjustment analysis for the primary observed-ASV exposure, showing changes in association estimates from unadjusted to demographic, clinical/socioeconomic, and diet/water fully adjusted models. (D) Rarefaction-depth sensitivity analysis for observed ASVs across 2,000, 4,000, 6,000, 8,000, and 10,000 reads. NHANES provided oral-only population-based context under measured covariate adjustment and was not used as direct validation of internal salivary genus-level candidates. ASV, amplicon sequence variant; CI, confidence interval; FDR, false-discovery rate; OR, odds ratio.


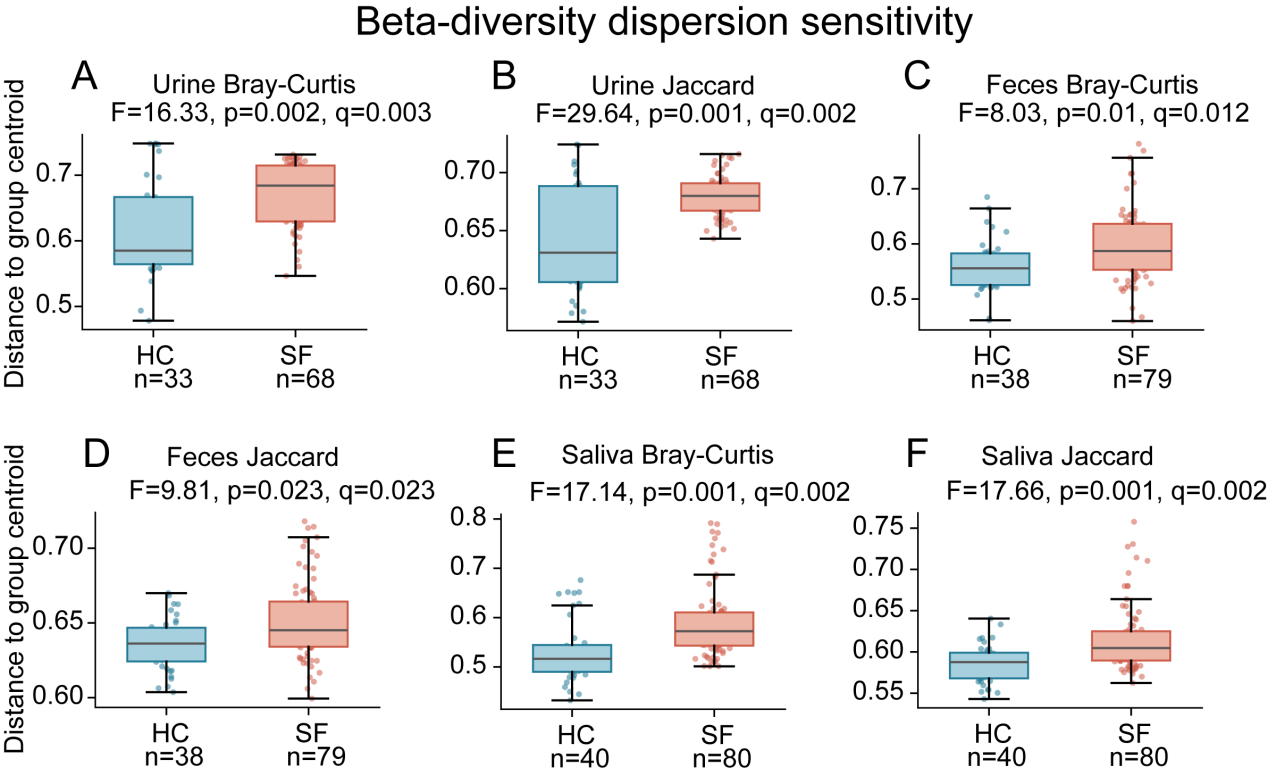


**Supplementary Figure S3. Beta-diversity dispersion sensitivity analysis.** (A) Urinary Bray-Curtis distance-to-centroid comparison between HC and SF samples, with PERMDISP F statistic, raw p value, and FDR-adjusted q value displayed above the panel. (B) Urinary Jaccard distance-to-centroid comparison between HC and SF samples. (C) Fecal Bray-Curtis distance-to-centroid comparison between HC and SF samples. (D) Fecal Jaccard distance-to-centroid comparison between HC and SF samples. (E) Salivary Bray-Curtis distance-to-centroid comparison between HC and SF samples. (F) Salivary Jaccard distance-to-centroid comparison between HC and SF samples. These analyses support interpretation of beta-diversity findings as community-distribution differences with a dispersion component rather than complete SF-HC separation. FDR, false-discovery rate; HC, healthy controls; SF, stone formers.


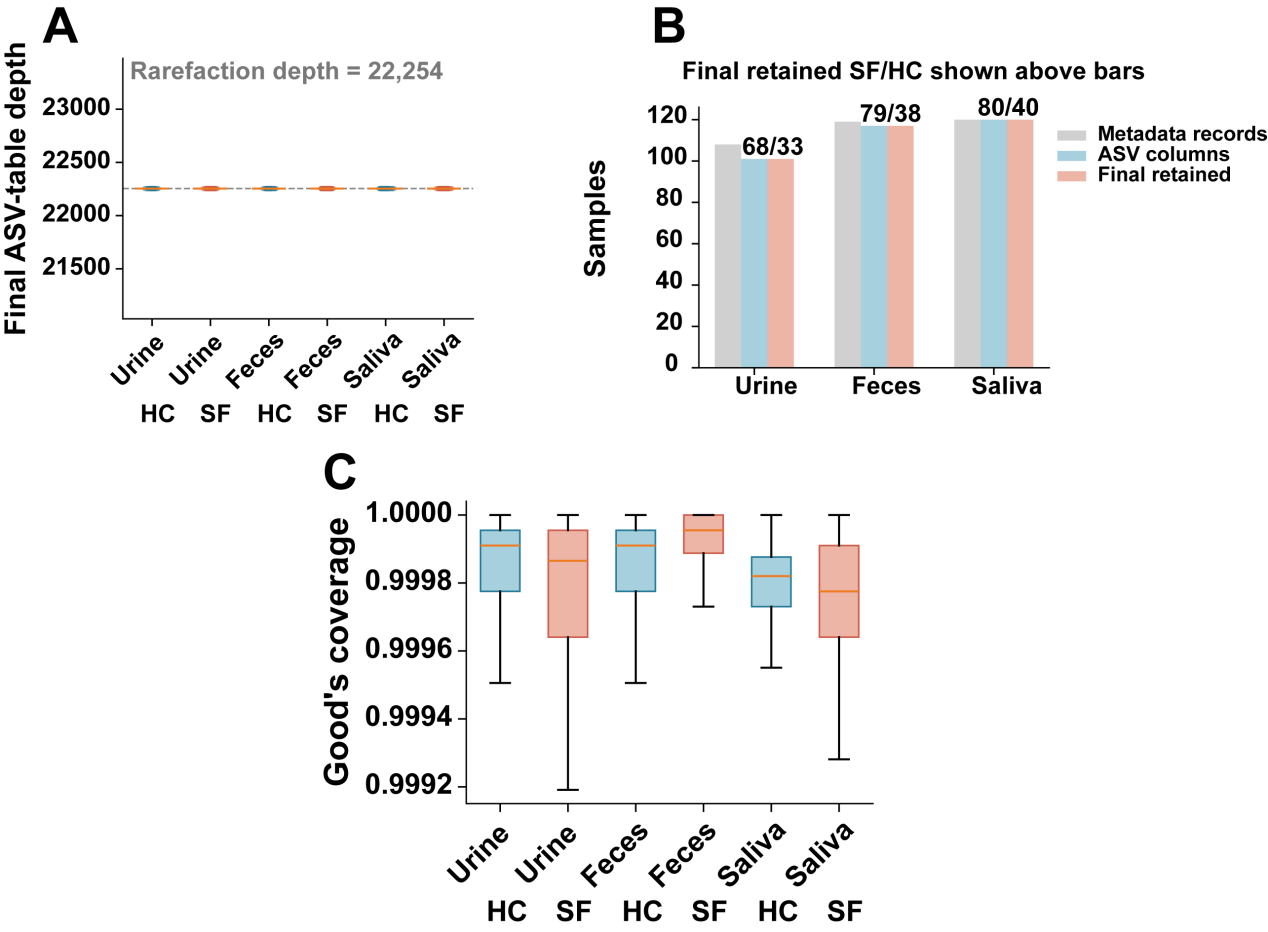


**Supplementary Figure S4. Sequencing depth, rarefaction eligibility, and sample-level quality control.** (A) Sequencing-depth distributions of available final ASV-table samples by habitat and group. The dashed horizontal line indicates the fixed rarefaction depth of 22,254 reads used for alpha- and beta-diversity analyses. (B) Comparison of metadata records, ASV-table sample columns, and final retained samples by habitat; final SF and HC sample counts are shown above the corresponding bars. (C) Good's coverage distributions by habitat and group after construction of the final analysis table. These quality-control summaries document sample retention, rarefaction eligibility, and sequencing-coverage adequacy for the internal analyses. ASV, amplicon sequence variant; HC, healthy controls; SF, stone formers.


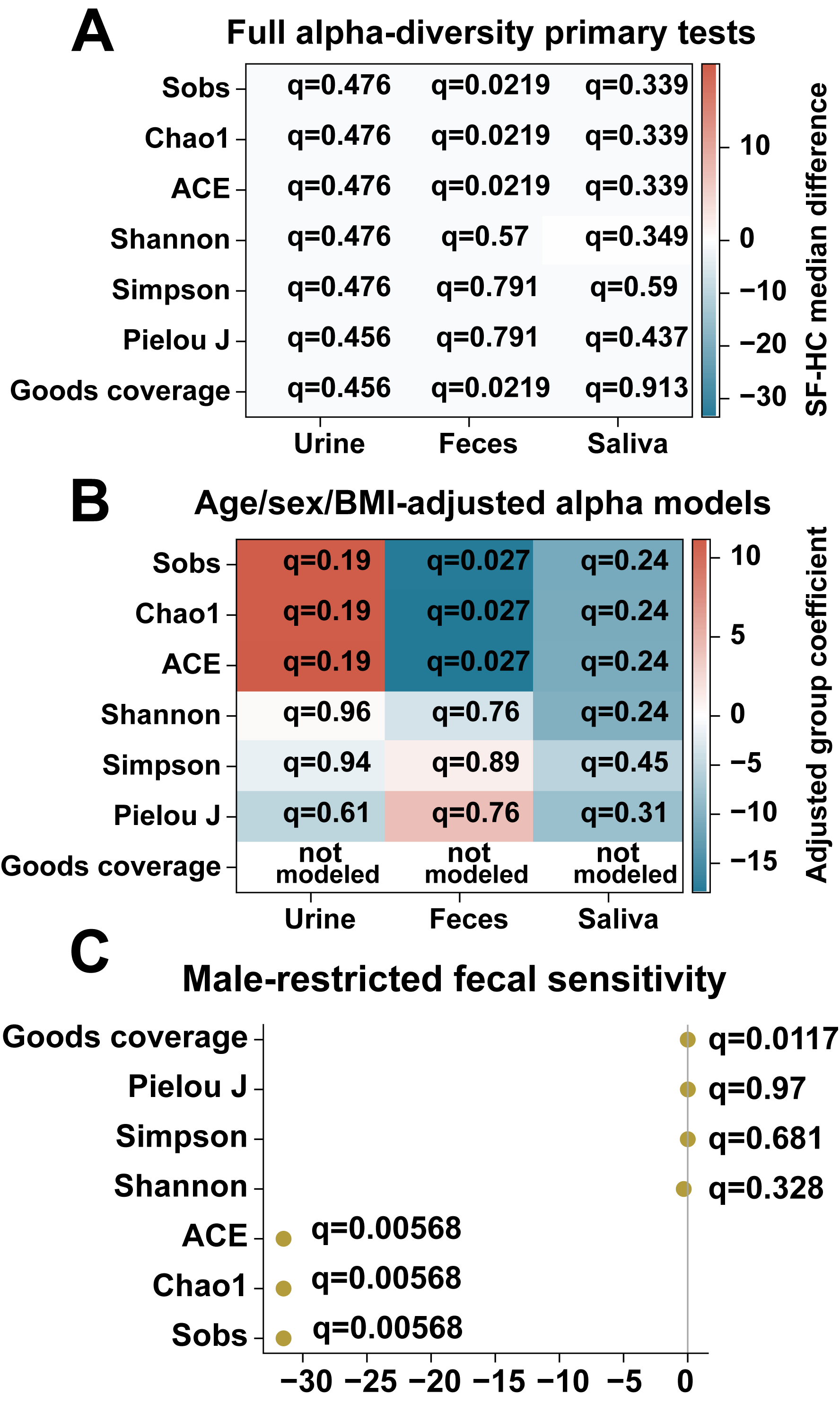


**Supplementary Figure S5. Alpha-diversity sensitivity analyses.** (A) Heatmap of primary SF-HC median differences for seven alpha-diversity metrics across urinary, fecal, and salivary habitats. Cell labels indicate within-habitat FDR-adjusted q values from two-sided Mann-Whitney U tests. (B) Age-, sex-, and BMI-adjusted rank-based sensitivity models for the same alpha-diversity metrics. Heatmap color indicates the adjusted group coefficient, and cell labels show FDR-adjusted q values within the prespecified testing family. (C) Male-restricted fecal alpha-diversity sensitivity analysis, showing SF-HC median differences and within-site FDR-adjusted q values for each metric. These analyses assess whether the fecal richness signal is robust to alternative correction and covariate-sensitivity frameworks. BMI, body mass index; FDR, false-discovery rate; HC, healthy controls; SF, stone formers.


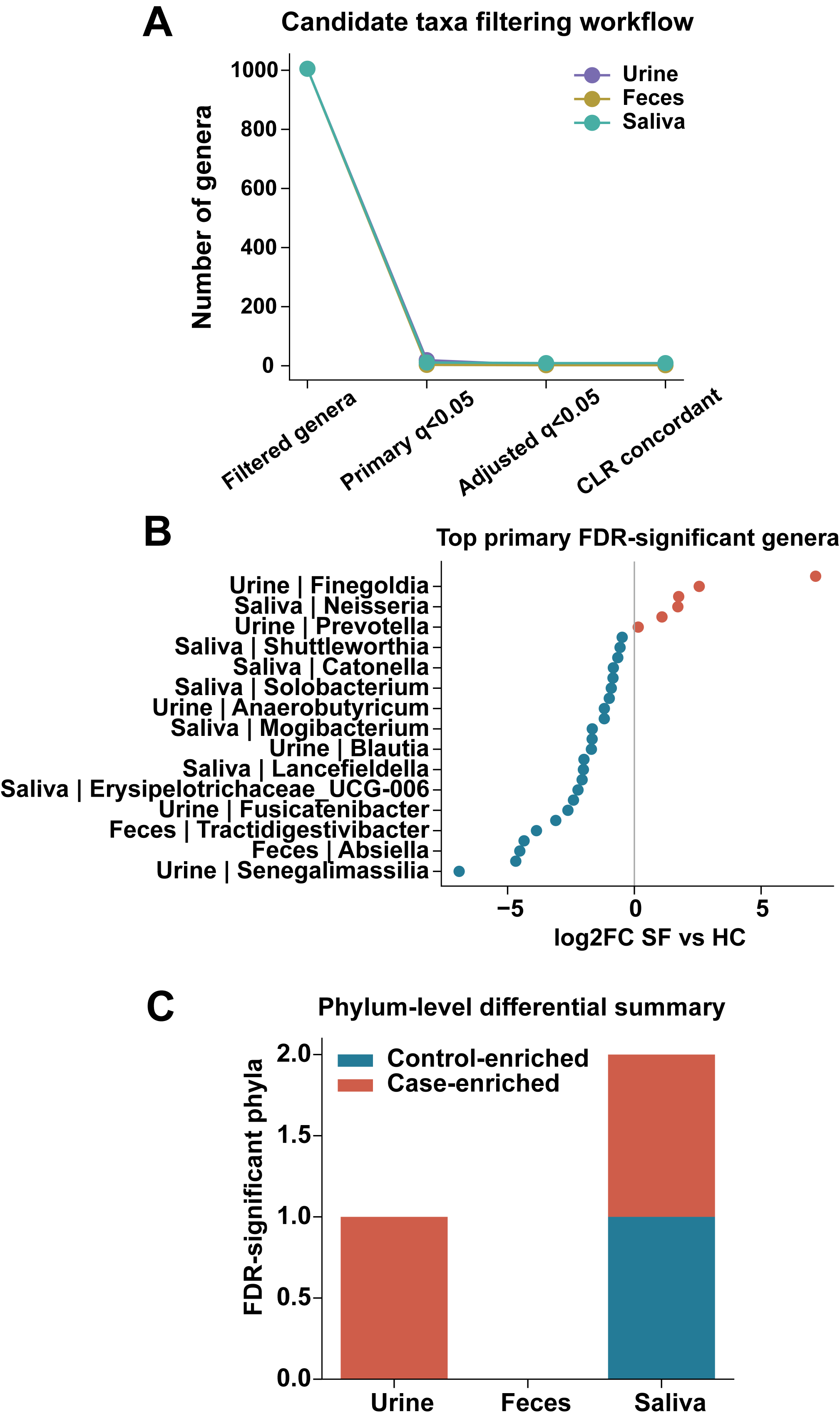


**Supplementary Figure S6. Taxonomic robustness and candidate-genera classification.** (A) Candidate-taxa filtering workflow by habitat, showing the number of filtered genera, primary FDR-significant genera, genera remaining significant after age/sex/BMI adjustment, and genera with concordant CLR-based compositional sensitivity support. (B) Effect-size overview of the top primary FDR-significant genus-level differences. Points indicate log2 fold change for SF versus HC; positive values indicate SF enrichment and negative values indicate HC enrichment. (C) Phylum-level differential summary by habitat and enrichment direction. Genera meeting primary FDR significance, adjusted-model FDR significance, concordant directionality, and CLR-based sensitivity support were classified as internally stable candidate genera. BMI, body mass index; CLR, centered log-ratio; FDR, false-discovery rate; HC, healthy controls; SF, stone formers.


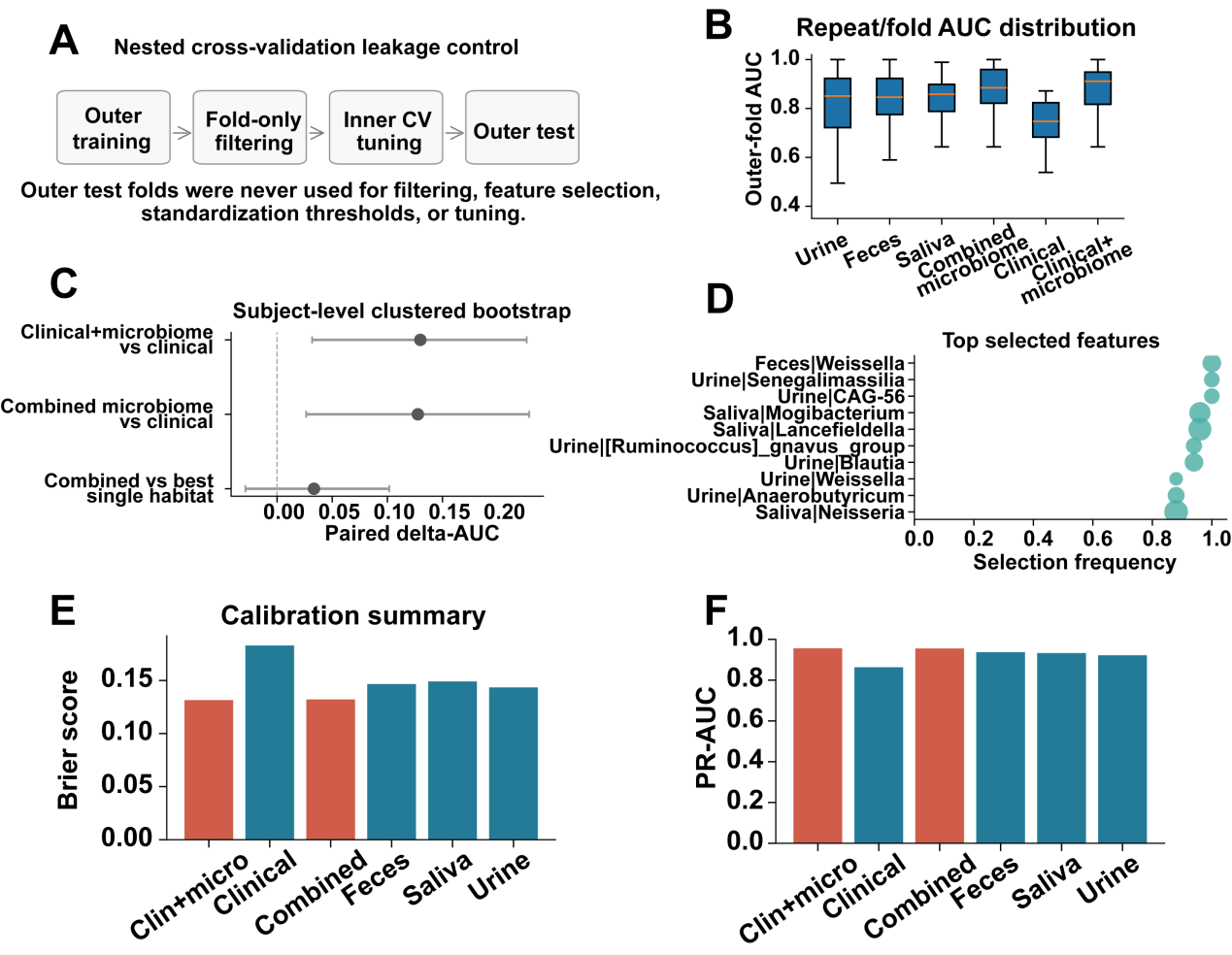


**Supplementary Figure S7. Random forest stability under repeated nested cross-validation.** (A) Schematic of leakage control. Feature filtering, feature selection, preprocessing decisions, and hyperparameter tuning were restricted to outer-training data and inner cross-validation loops; outer-test folds were reserved for performance estimation. (B) Distribution of outer-fold AUC values across repeated nested cross-validation for urine-only, feces-only, saliva-only, combined microbiome, clinical baseline, and clinical-plus-microbiome matched-subject models. (C) Paired delta-AUC comparisons estimated using subject-level clustered bootstrap resampling to account for repeated predictions from the same participant. (D) Top selected features in the combined microbiome model, displayed by selection frequency across outer training folds; point size reflects mean importance among folds in which the feature was selected. (E) Calibration summary using Brier scores for matched-subject models, with lower values indicating better average probabilistic calibration. (F) Precision-recall summary using average-precision PR-AUC for the same matched-subject models. AUC, area under the receiver operating characteristic curve; PR-AUC, area under the precision-recall curve.


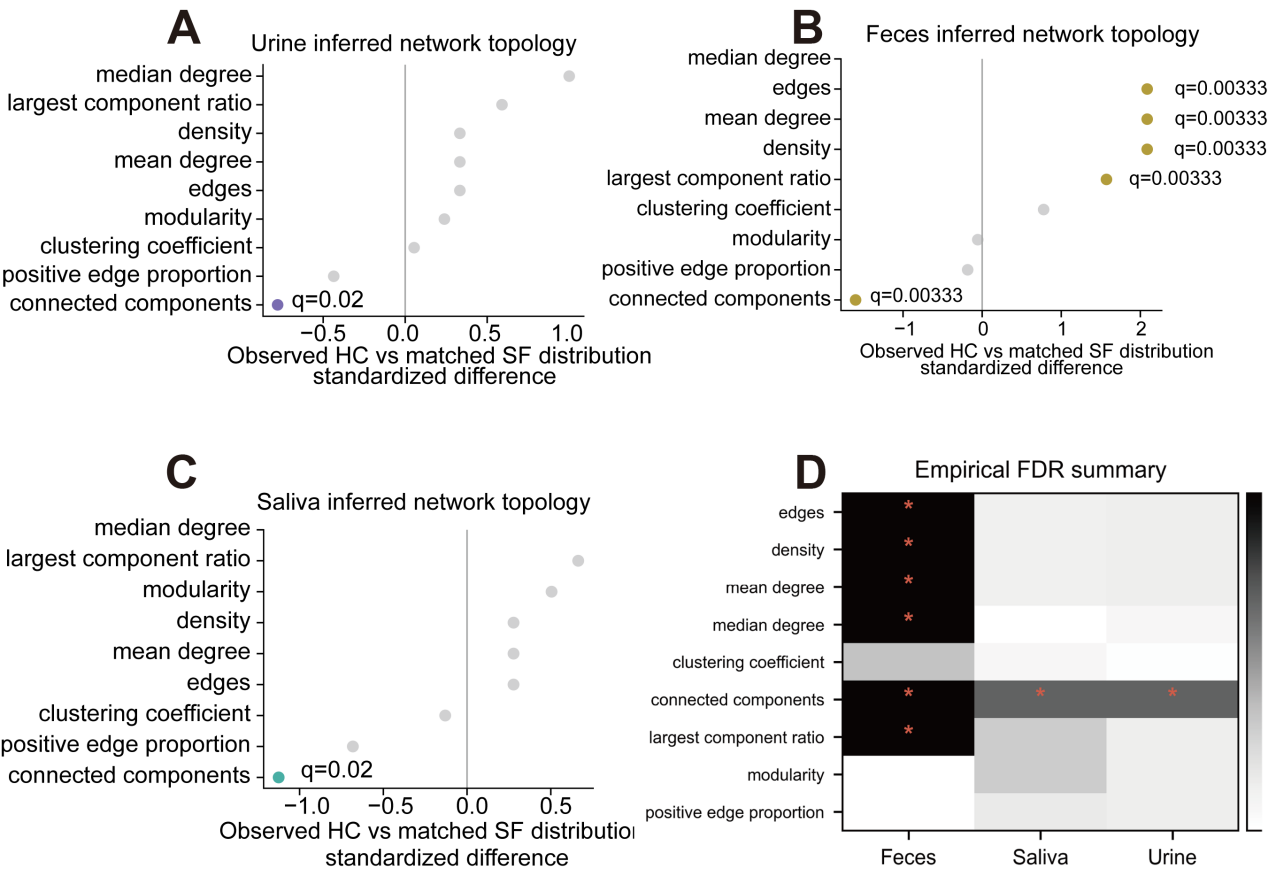


**Supplementary Figure S8. Sample-size matched inferred co-occurrence network topology sensitivity analysis.** (A) Urinary inferred-network topology summary. Points show standardized differences between observed HC topology metrics and the matched SF resampling distribution; highlighted points indicate metrics meeting empirical FDR significance. (B) Fecal inferred-network topology summary using the same matched-resampling framework. (C) Salivary inferred-network topology summary using the same matched-resampling framework. (D) Empirical FDR summary across prespecified topology metrics and habitats. Asterisks mark topology metrics that remained significant after empirical FDR correction. These network analyses are exploratory and describe inferred co-occurrence topology, not direct microbial interactions. FDR, false-discovery rate; HC, healthy controls; SF, stone formers.


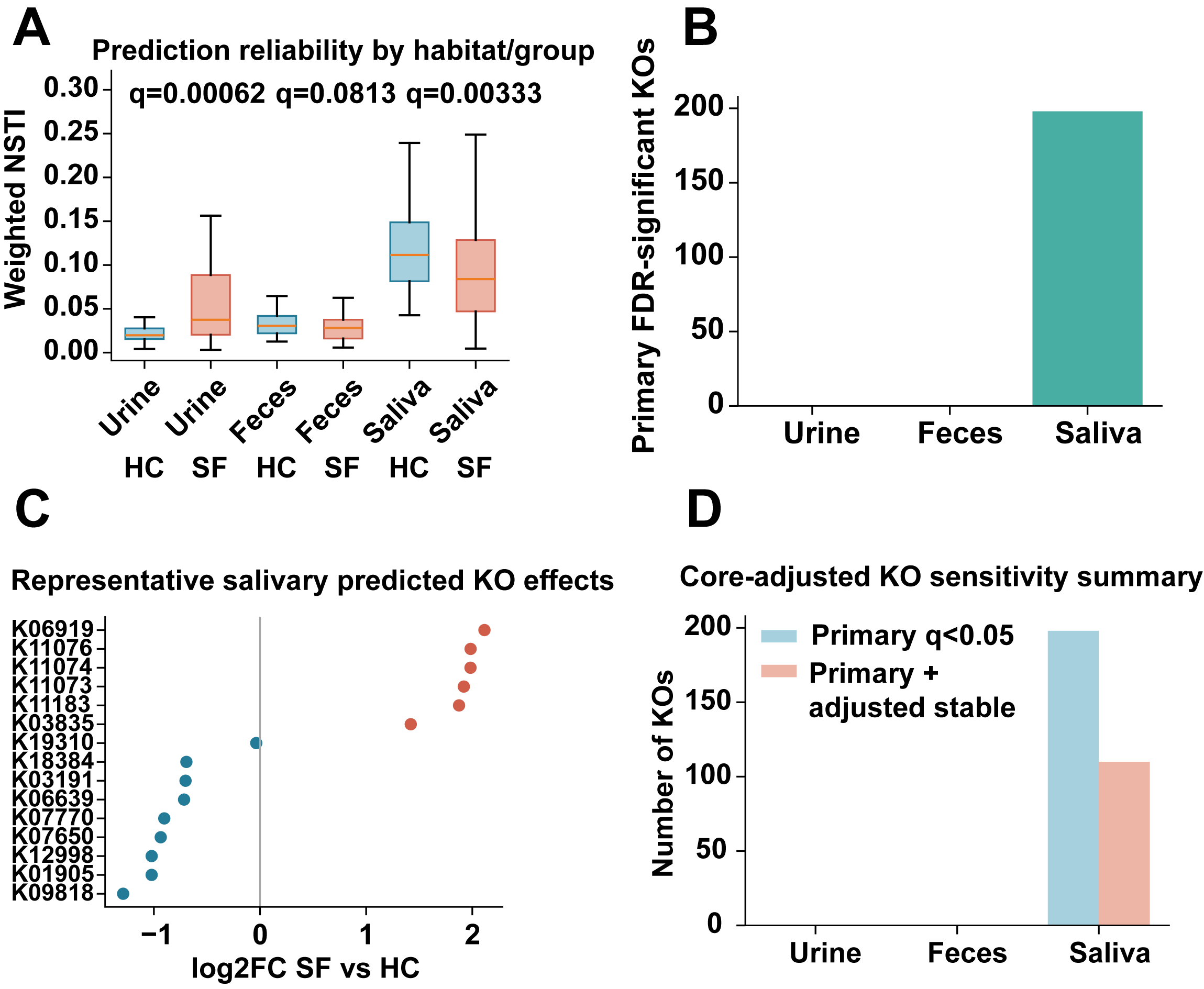


**Supplementary Figure S9. PICRUSt2 predicted functional-potential and NSTI reliability analysis.** (A) Sample-level weighted nearest sequenced taxon index (NSTI) distributions by habitat and group. FDR-adjusted q values summarize within-habitat group comparisons; higher or group-differential NSTI values indicate greater caution in interpreting 16S-based predicted functional profiles. (B) Number of primary site-wise FDR-significant predicted KEGG Orthology (KO) features by habitat. (C) Representative salivary predicted KO effect sizes displayed as log2 fold change for SF versus HC; positive values indicate SF-enriched predicted KO abundance and negative values indicate HC-enriched predicted KO abundance. (D) Core-adjusted KO sensitivity summary comparing primary FDR-significant KOs with KOs that remained significant and directionally stable after age/sex/BMI adjustment. PICRUSt2 results represent predicted functional potential and require direct functional validation. BMI, body mass index; FDR, false-discovery rate; HC, healthy controls; KO, KEGG Orthology; NSTI, nearest sequenced taxon index; SF, stone formers.


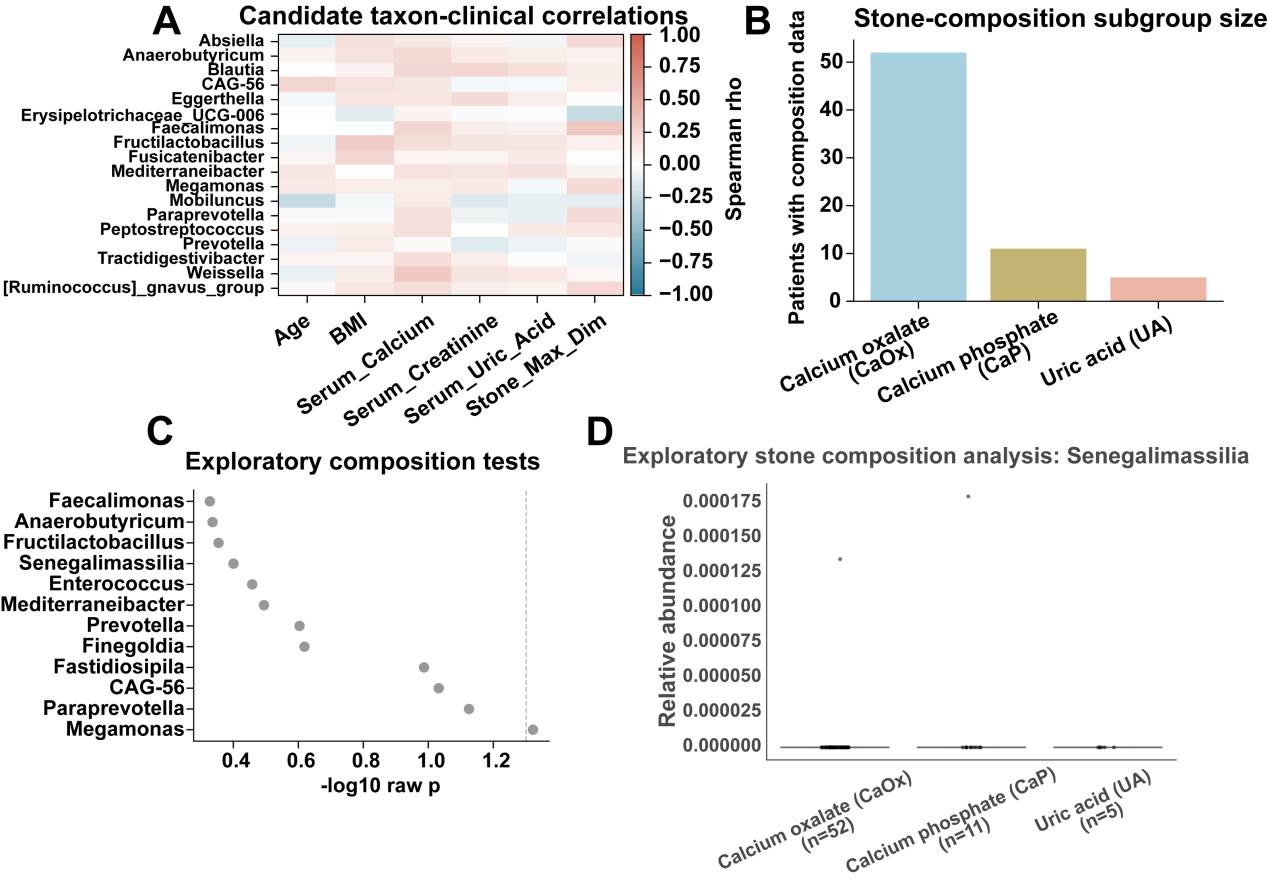


**Supplementary Figure S10. Exploratory clinical-phenotype and stone-composition analyses.** (A) Heatmap of Spearman correlations between candidate genera and recorded clinical phenotypes among SF. Asterisks indicate associations meeting FDR significance within the prespecified testing family, where applicable. (B) Stone-composition subgroup sample sizes among patients with available composition data, illustrating the limited effective sample size for composition-stratified microbial comparisons. (C) Exploratory stone-composition subgroup tests for urinary candidate genera, ranked by raw p value for descriptive visualization. Formal interpretation was based on prespecified FDR-corrected overall tests reported in the supplementary table. (D) Exploratory stone-composition subgroup analysis of urinary Senegalimassilia relative abundance across calcium oxalate (CaOx), calcium phosphate (CaP), and uric acid (UA) stone subtypes among stone formers with available composition metadata and detectable genus-level profiles. Individual data points are overlaid on boxplots. Given small subgroup sizes and limited statistical power, these analyses are descriptive and do not support composition-specific microbiome conclusions. CaOx, calcium oxalate; CaP, calcium phosphate; FDR, false-discovery rate; SF, stone formers; UA, uric acid.


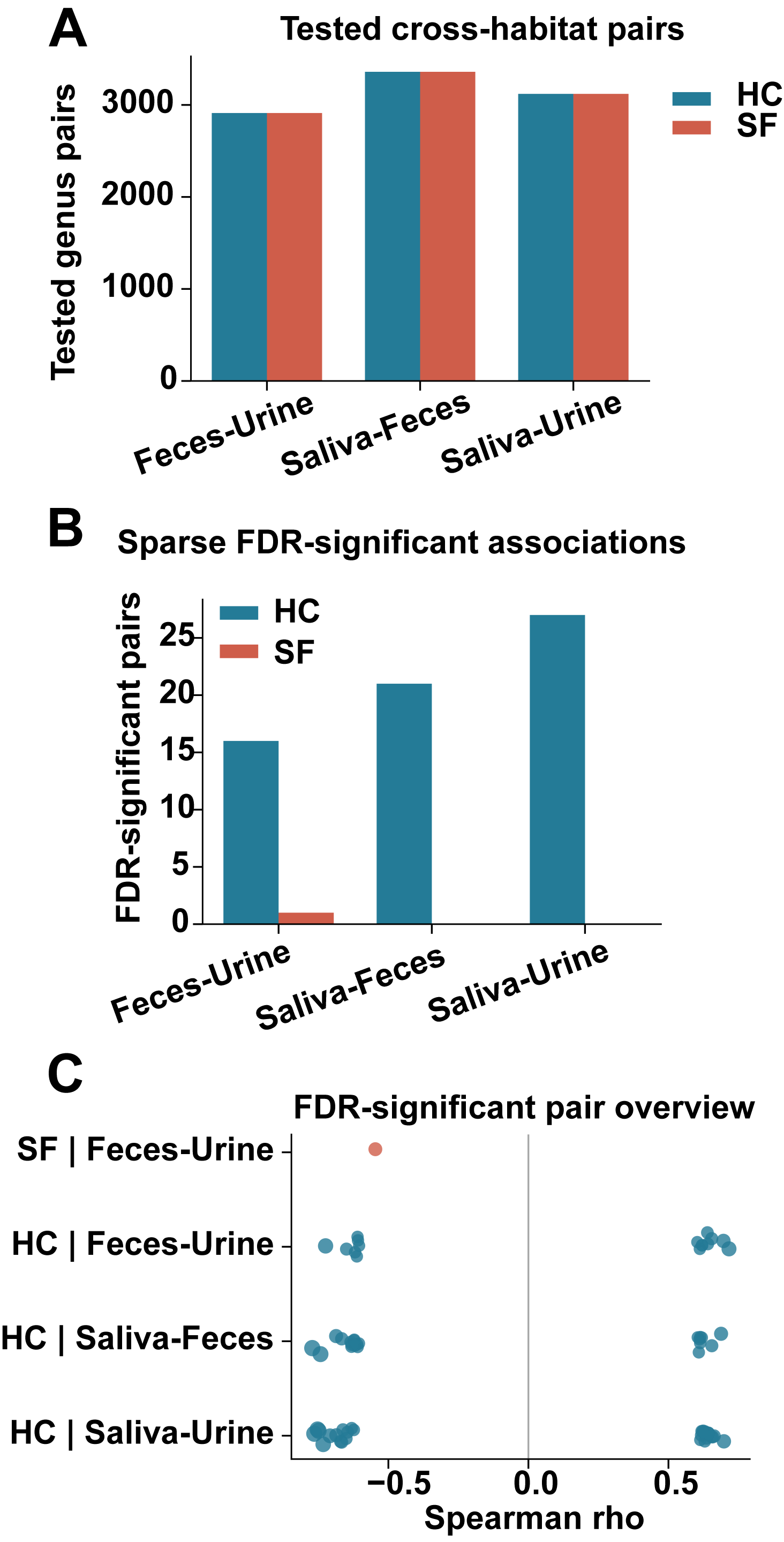


**Supplementary Figure S11. Cross-habitat genus-level association sparsity analysis.** (A) Number of tested cross-habitat genus pairs for feces-urine, saliva-feces, and saliva-urine comparisons in HC and SF groups. (B) Number of genus pairs passing FDR correction in each cross-habitat comparison and group. (C) Overview of FDR-significant cross-habitat genus pairs. The x-axis shows Spearman correlation coefficients, and points are stratified by group and habitat pair. These analyses were performed in matched participants and show sparse cross-habitat covariation rather than a consistent SF-specific oral-gut-urinary coordination pattern. FDR, false-discovery rate; HC, healthy controls; SF, stone formers.


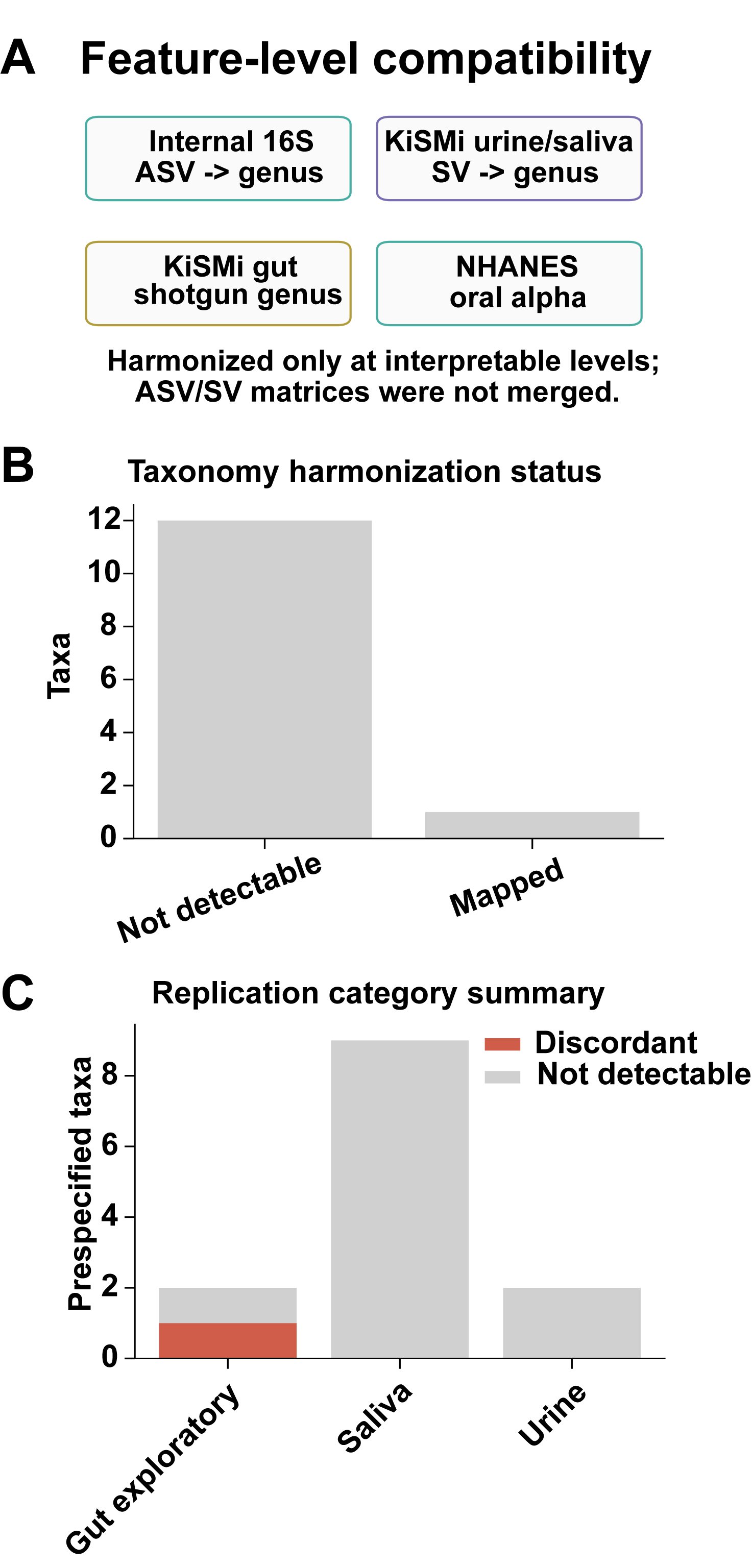


**Supplementary Figure S12. External-data harmonization and evidence-boundary summary.** (A) Feature-level compatibility schematic. Internal 16S ASVs, KiSMi urine/saliva sequence variants, KiSMi gut shotgun genera, and NHANES oral alpha-diversity metrics were harmonized only at interpretable analysis levels; ASV/sequence-variant matrices were not merged across studies. (B) Taxonomy harmonization status for prespecified internal candidate taxa, showing the number of taxa that could be mapped or were not detectable/evaluable in the external matrices. (C) Replication-category and evidence-boundary summary across urinary, salivary, exploratory gut, and NHANES oral panels. These external analyses define contextual support and interpretive limits; they do not establish causality, do not validate a broad cross-habitat coordination mechanism, and do not replace harmonized prospective external validation. ASV, amplicon sequence variant; HC, healthy controls; NHANES, National Health and Nutrition Examination Survey; SF, stone formers.
